# Potential antiviral effects of pantethine against SARS-CoV-2

**DOI:** 10.1038/s41598-023-29245-0

**Published:** 2023-02-08

**Authors:** M. Abou-Hamdan, R. Saleh, S. Mani, P. Dournaud, M. Metifiot, M. L. Blondot, M. L. Andreola, F. Abdel-sater, M. De Reggi, P. Gressens, M. Laforge

**Affiliations:** 1 NeuroDiderot, Inserm, Université Paris Cité, 48 Boulevard Sérurier, 75019 Paris, France; 2 Biology Department, Faculty of Sciences (I), Lebanese University, Beirut, Lebanon; 3 Université Bordeaux, CNRS, UMR 5234, Microbiologie Fondamentale et Pathogénicité, 33076 Bordeaux, France; 4 Biochemistry Department, Faculty of Sciences (I), Lebanese University, Beirut, Lebanon

**Keywords:** Drug discovery, Immunology, Diseases, Medical research

## Abstract

SARS-CoV-2 interacts with cellular cholesterol during many stages of its replication cycle. Pantethine was reported to reduce total cholesterol levels and fatty acid synthesis and potentially alter different processes that might be involved in the SARS-CoV-2 replication cycle. Here, we explored the potential antiviral effects of pantethine in two in vitro experimental models of SARS-CoV-2 infection, in Vero E6 cells and in Calu-3a cells. Pantethine reduced the infection of cells by SARS-CoV-2 in both preinfection and postinfection treatment regimens. Accordingly, cellular expression of the viral spike and nucleocapsid proteins was substantially reduced, and we observed a significant reduction in viral copy numbers in the supernatant of cells treated with pantethine. In addition, pantethine inhibited the infection-induced increase in TMPRSS2 and HECT E3 ligase expression in infected cells as well as the increase in antiviral interferon-beta response and inflammatory gene expression in Calu-3a cells. Our results demonstrate that pantethine, which is well tolerated in humans, was very effective in controlling SARS-CoV-2 infection and might represent a new therapeutic drug that can be repurposed for the prevention or treatment of COVID-19 and long COVID syndrome.

## Introduction

Emerging SARS-CoV-2 variants can weaken the antiviral immune response. In addition to their ability to escape the immune system and their characteristics of being more contagious and deadlier^1,2^, they can also escape antibody neutralization^3^. Moreover, highly pathogenic SARS-CoV-2 variants that emerge in the future could dampen T-cell surveillance. Such threats highlight the need for drug-based alternative therapeutic strategies. Our aim is to take advantage of pantethine, a well-known drug with multiple known mechanisms of action, to develop a new antiviral therapy.

The low-molecular-weight thiol pantethine is the major precursor of coenzyme A, a cofactor in over 70 enzymatic pathways in the body. It reduces total cholesterol levels and total fatty acid synthesis^4,5^ and alters the lipid composition and cholesterol content of cell membrane rafts^6^. Host cholesterol and lipid rafts are important for virus entry into permissive cells. ACE2, the main host receptor involved in virus entry, is localized in lipid rafts^7,8^, and syncytium formation mediated by viral spike-ACE2 fusion occurs specifically in cholesterol-rich regions^9^. In addition, host cholesterol is important for subsequent stages of the SARS-CoV-2 replication cycle, e.g., activation, internalization, egression, and cell-to-cell transmission^8,10–13^. Indeed, the depletion of cholesterol from cell membranes significantly was reported to reduce the infectivity of SARS-CoV^7,14^, a close relative of SARS-CoV-2^15^. Furthermore, dyslipidemia is a known risk factor for cardiovascular disease, one of the common symptoms of long COVID syndrome^16^. An abnormal cholesterol level was associated with increased mortality and severity of COVID-19^17–19^, which is reported to be a risk factor associated with long COVID^16,20^.

On the other hand, a decrease in thiol levels was proposed to be a molecular marker for increased risks of infection and development of serious COVID-19^21^. Several studies have indicated that the capacity of enveloped viruses to infect host cells depends on the precise thiol/disulfide balance in their surface glycoprotein complexes, and any perturbations in this redox state affect virus/cell interactions^15–22^. In addition, it has been proposed that thiols can impair the binding of SARS-CoV-2 spike protein to its receptor ACE2 by reducing the disulfide groups of this receptor to sulfhydryl (SH) groups^23^. In support of this hypothesis, a recent study found that thiol-based drugs decreased the binding of the SARS-CoV-2 spike protein to its receptor ACE2, impaired the entry efficiency of SARS-CoV-2 spike pseudotyped virus, and inhibited SARS-CoV-2 live virus infection^24^. Furthermore, disulfide bond formation between Cys residues was implicated in the association between the SARS-CoV-2 E and S proteins, which are involved in important aspects of the viral replication cycle^25,26^.

Moreover, a recent study listed pantethine among the FDA-approved drugs that could potentially bind to the substrate-binding site of the main protease of SARS-CoV-2 (M^pro^) and inhibit its activity^27^, which was proposed as a key antiviral drug target^28^. These potential effects of pantethine on different processes important for SARS-CoV-2 pathogenicity prompted us to explore the antiviral effect of pantethine against SARS-CoV-2, which has never been studied before, in two in vitro models of SARS-CoV-2 infection, in Vero E6 and in Calu-3a cells.

In this work, we report a significant antiviral effect of pantethine in Vero E6 and Calu-3a cells infected with SARS-CoV-2. Our results show that pantethine exerts its effects on viral entry under long-drug incubation condition prior to viral infection and more effectively on postentry and full-time treatment condition suggesting an interference of pantethine with the viral cycle. The protective effect of pantethine was associated with a reduction in the infection-induced increases in the expression levels of TMPRSS2 and different HECT E3 ligases, which were proposed to be potential therapeutic targets for SARS-CoV-2 infection. As a result of the decrease in intracellular viral replication, the interferon beta response and the inflammatory response disappeared in Calu-3a cells.

## Results

### Pantethine was not toxic to Vero E6 cell cultures

The toxicity of pantethine to Vero E6 cells was evaluated with Viability 405/452 Fixable Dye (Miltenyi Biotec). Vero E6 cells that were infected or not with SARS-CoV-2 (BetaCoV/France/IDF0372/2020) were treated with different concentrations of pantethine (250–2000 µM) for 72 h before incubation with the dye. No toxicity was observed at the different doses of pantethine used for 72 h of treatment in all studied conditions (Supplementary Fig. S1).

### Pantethine reduced SARS-CoV-2 infection in Vero E6 cell cultures

The efficacy of pantethine as an antiviral treatment was studied in Vero E6 cells infected with SARS-CoV-2 at different stages of virus infection (full-time treatment, postentry treatment, and preentry treatment with short and long incubations). For pantethine treatments, the concentrations of 50, 100, 250, 500 and 1000 µM were used based on previous studies using pantethine on different cell types with no toxic effects^6,29,30^. Indeed, these concentrations are below the concentration of 2000 µM of pantethine tested in our experimental model with nontoxic effect (Supplementary Fig. S1). We evaluated the variation in the SARS-CoV-2 infection rate by detecting the viral spike (S) protein in Vero E6 cells by flow cytometry analysis (live intracellular monitoring of the infection) and by detecting viral S and nucleocapsid (N) protein expression in Vero E6 cells by western blot analysis. We also quantified the viral copy numbers in PBS-washed cells (intracellular quantification) and in cell supernatants through quantitative real-time RT-PCR for viral nucleocapsid (*N*) and nonstructural protein 6 (*NSP6*) genes. Remdesivir (5 and 10 µM) was used as a positive control for antiviral efficacy because it inhibits the viral RNA-dependent RNA polymerase (RdRp).

**Figure 1:**
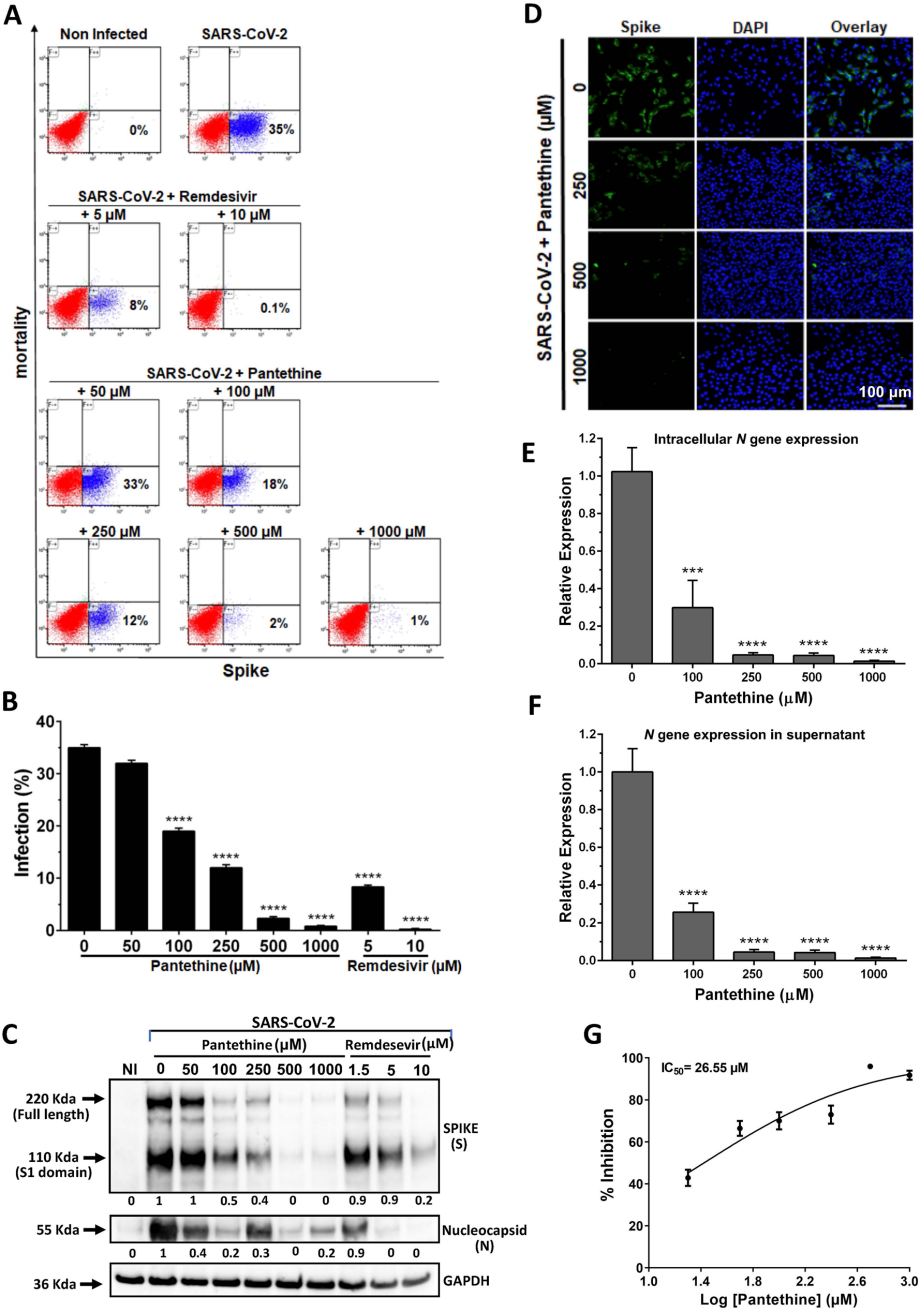
Full-time pantethine treatment reduced SARS-CoV-2 infection in Vero E6 cell cultures. Full-time treatment with pantethine or remdesivir reduced the infection of Vero E6 cells with SARS-CoV-2 (MOI 0.05) significantly and in a dose-dependent manner. (**A**) Seventy-two hours post-infection, cells were collected and stained to determine viability and infection rates through the detection of the viral spike (S) protein in cells by flow cytometry analysis. The presented data, with the percentage of infection in each plot, are representative of 1 of 3 independent experiments that yielded similar results. (**B**) Data represent the percentage of infection observed with the flow cytometry analysis experiments and are shown as the mean + SEM of results obtained from 3 independent experiments with 3 independent points per condition. (**C**) Seventy-two hours post-infection, cells were lysed with RIPA buffer, and western blot analyses were performed to detect the expression of the viral spike (S), full-length S1 domain, and nucleocapsid (N) proteins. GAPDH was used as a loading control. The numbers below the blots represent relative expression levels. For each viral protein, the levels of the uninfected cells were set at 1. (**D**) Immunofluorescence microscopy of virus infection upon treatment with pantethine. Infected cells treated or not treated with pantethine were fixed and analyzed by confocal microscopy for the detection of the viral S protein; *scale bars* = 100 μm. Virus yields in infected cells (**E**) and in their supernatants (**F**) were quantified by qRT-PCR for the viral *N* gene. Calculated Ct values were converted to the fold-reduction of samples compared to the housekeeping gene *GAPDH* (for cells) or to noninfected cells (for supernatants) using the ΔΔCt method (fold change in viral RNA = 2^−ΔΔCt^). In (**B**), (**E**) and (**F**), the results represent the mean + SEM. (**G**) An inhibitory dose–response curve based on viral *N* gene expression in supernatants was used to determine the IC50 using GraphPad Prism software. The results represent the mean ± SEM. In all experiments, the results were obtained from 4 independent experiments with 3 independent points per condition. ***p < 0.001 and ****p < 0.0001 compared to the control group (infected untreated cells) by one-way ANOVA followed by Dunnett's post hoc test.

For full-time treatment, Vero E6 cells were pretreated with different concentrations of pantethine for 1 h prior to virus infection, followed by incubation with the virus in the presence of pantethine. The virus and pantethine were maintained in the culture until the end of the experiment. Treatment with pantethine at different concentrations (100–1000 µM) reduced the infection of Vero E6 cells by SARS-CoV-2 significantly and in a dose-dependent manner (Fig. 1 and Supplementary Fig. S2). The number of Vero E6 cells positive for the viral S protein was significantly reduced (by 52.3, 70.2, 92, and 97.1%) by pantethine treatment at concentrations of 100, 250, 500 and 1000 µM, respectively (Fig. 1A,B). Indeed, the expression of the viral S and N proteins was reduced in cells treated with pantethine (Fig. 1C). These observations were confirmed by visualization of viral S protein expression through immunofluorescence microscopy at 72 h post-infection. (Fig. 1D). Accordingly, we observed a significant reduction in viral *N* and *NSP6* gene expression within cells (Fig. 1E and Supplementary Fig. S6A) and in the supernatant (Fig. 1F and Supplementary Fig. S6B) in cultures treated with pantethine, with a calculated IC50 of 26.55 µM (Fig. 1G). The observed reductions after pantethine treatments were comparable to those achieved with remdesivir treatments (Fig. 1A–C and Supplementary Fig. S7A).

**Figure 2:**
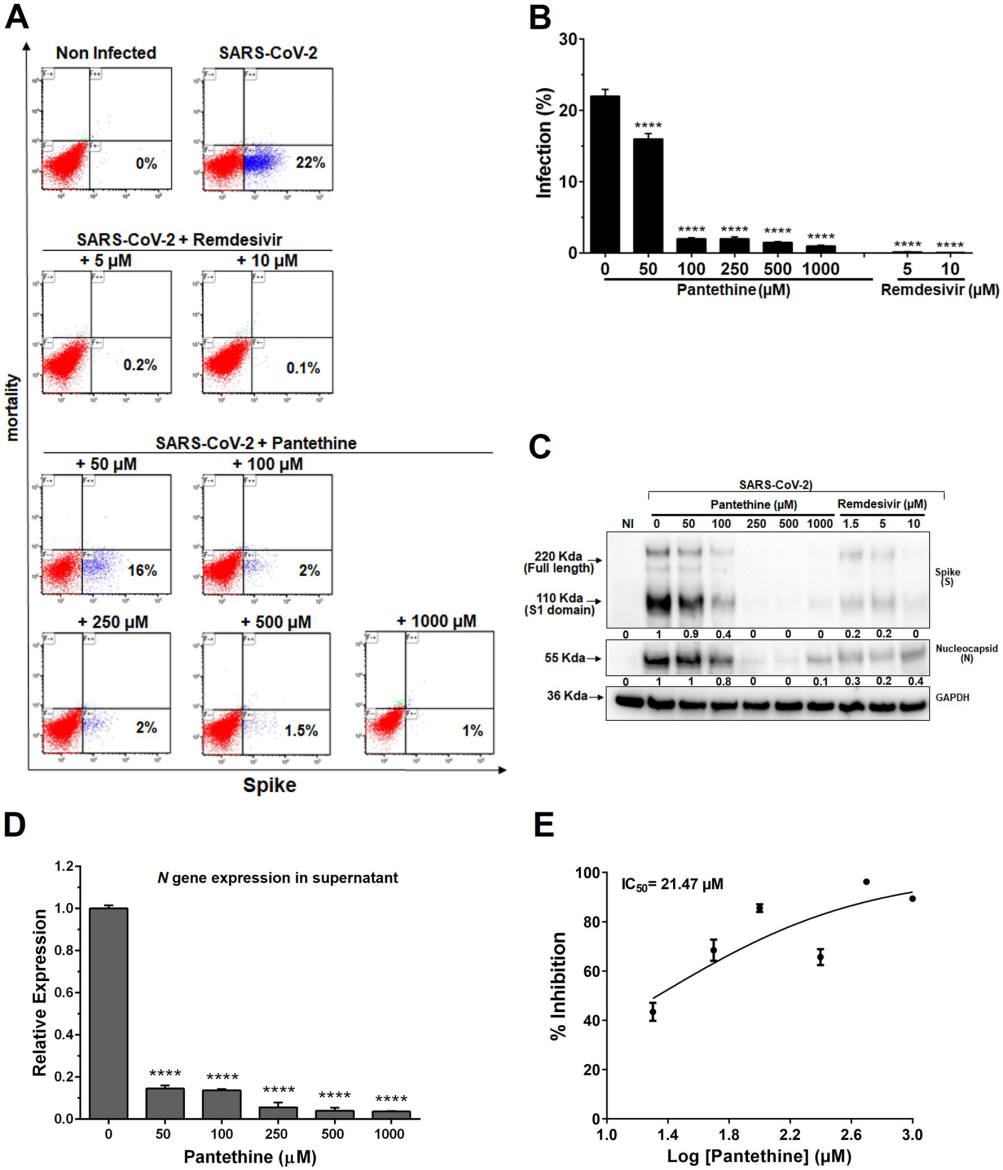
Pantethine postentry treatment reduced SARS-CoV-2 infection in Vero E6 cell cultures. Post-entry treatment with pantethine or remdesivir reduced the infection of Vero E6 cells by SARS-CoV-2 (MOI 0.05) significantly and in a dose-dependent manner. (**A**) Seventy-two hours post-infection, cells were collected and stained to determine viability and infection rates through the detection of the viral spike (S) protein in cells by flow cytometry analysis. The presented data, with the percentage of infection in each plot, are representative of 1 of 3 independent experiments that yielded similar results. (**B**) Data represent the percentage of infection observed with the cytometry-analysis experiments and are shown as the mean + SEM of results obtained from 3 independent experiments with 3 independent points per condition. (**C**) Seventy-two hours post-infection, cells were lysed with RIPA buffer, and western blot analyses were performed to detect the expression of the viral spike (S), full-length S1 domain, and nucleocapsid (N) proteins. GAPDH was used as a loading control. The numbers below the blots represent relative expression levels. For each viral protein, the levels of the uninfected cells were set at 1. (**D**) Virus yields in the supernatant of infected cells were quantified by qRT-PCR for the viral *N* gene. Calculated Ct values were converted to fold-reduction of samples compared to the noninfected cells using the ΔΔCt method (fold change in viral RNA = 2^−ΔΔCt^). In (**B**) and (**D**), the results represent the mean + SEM. (**E**) An inhibitory dose–response curve based on viral *N* gene expression in supernatants was used to determine the IC50 using GraphPad Prism software. The results represent the mean ± SEM. In all experiments, the results were obtained from 4 independent experiments with 3 independent points per condition. ****p < 0.0001 compared to the control group (infected-untreated cells) by one-way ANOVA followed by Dunnett's post hoc test.

For postentry treatment, the virus was added to cells for 2 h, and then the virus-containing supernatant was replaced with drug-containing medium until the end of the experiment. Pantethine treatment significantly inhibited the infection rate (by 27% with 50 µM pantethine and by more than 97% with pantethine at concentrations of 100–1000 µM) (Fig. 2A,B). These results were in line with the significant reductions in viral S and N protein expression (Fig. 2C) and in *N* and *NSP6* gene expression in the supernatants of cells treated with pantethine (Fig. 2D and Supplementary Fig. S6C), with a calculated IC50 of 21.47 µM (Fig. 2E). These reductions were comparable to the effect of remdesivir treatments (Fig. 2A–C) and suggest that the effects of pantethine are involved in postentry pathways of SARS-CoV-2 pathogenicity.

**Figure 3:**
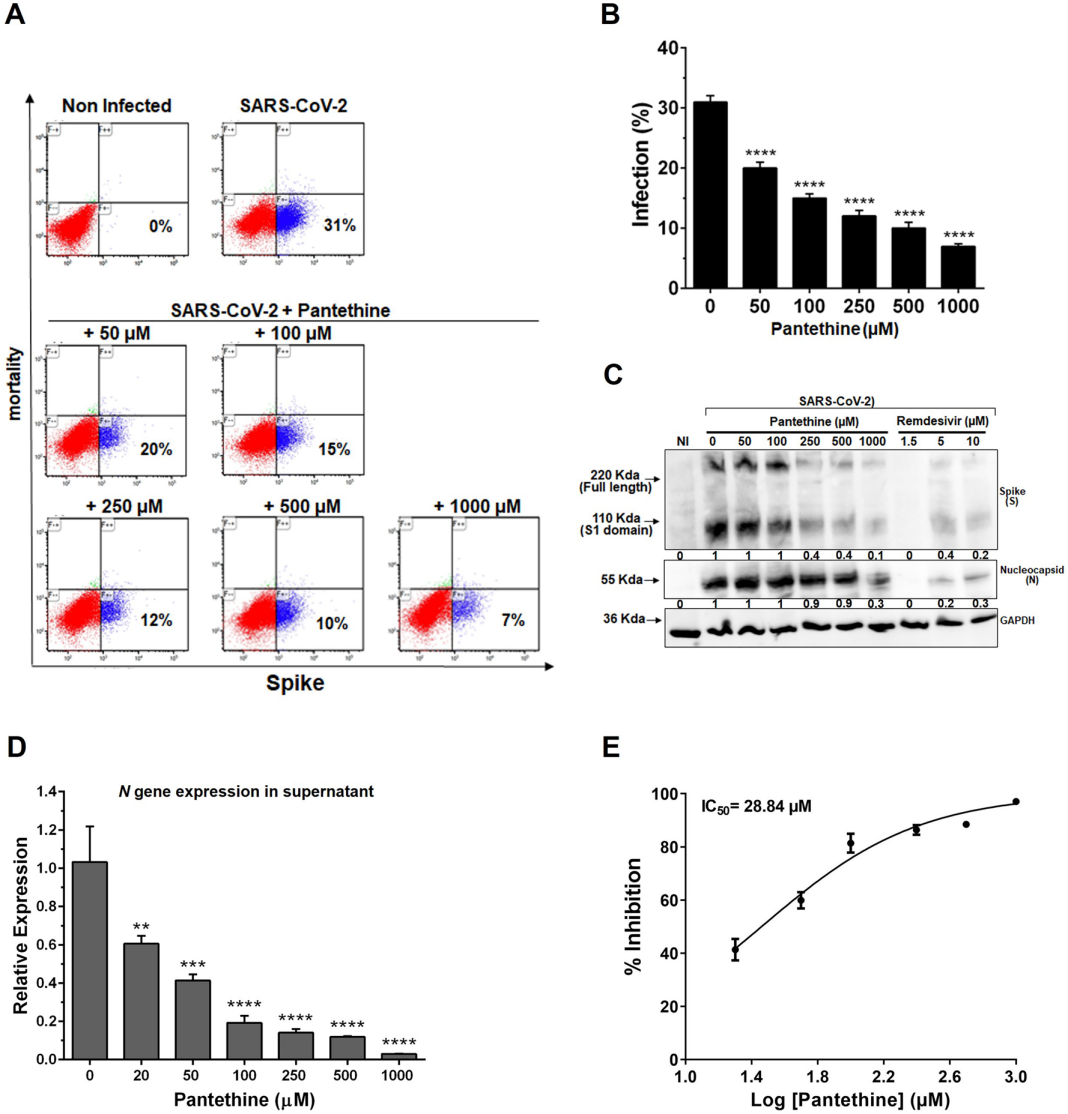
Pantethine preentry treatment reduced SARS-CoV-2 infection in Vero E6 cell cultures. Preentry treatment with pantethine reduced the infection of Vero E6 cells by SARS-CoV-2 (MOI 0.05) significantly and in a dose-dependent manner. (**A**) Seventy-two hours post-infection, cells were collected and stained to determine viability and infection rates through the detection of the viral spike (S) protein in cells by flow cytometry analysis. The presented data, with the percentage of infection in each plot, are representative of 1 of 3 independent experiments that yielded similar results. (**B**) Data represent the percentage of infection observed with the cytometry-analysis experiments and are shown as the mean + SEM of results obtained from 3 independent experiments with 3 independent points per condition. (**C**) Seventy-two hours post-infection, cells were lysed with RIPA buffer, and western blot analyses were performed to detect the expression of the viral spike (S), full-length S1 domain, and nucleocapsid (N) proteins. GAPDH was used as a loading control. The numbers below the blots represent relative expression levels. For each viral protein, the levels of the uninfected cells were set at 1. (**D**) Virus yields in the supernatant of infected cells were quantified by qRT-PCR for the viral *N* gene. Calculated Ct values were converted to fold-reduction of samples compared to the noninfected cells using the ΔΔCt method (fold change in viral RNA = 2^−ΔΔCt^). In (**B**) and (**D**), the results represent the mean + SEM. (**E**) An inhibitory dose–response curve based on viral *N* gene expression in supernatants was used to determine the IC50 using GraphPad Prism 8 software. The results represent the mean ± SEM. In all experiments, the results were obtained from 3 independent experiments with 3 independent points per condition. **p < 0.01, ***p < 0.001 and ****p < 0.0001 compared to the control group (infected untreated cells) by one-way ANOVA followed by Dunnett's post hoc test.

For preentry experiments, pantethine was added to cells for 1 or 24 h before virus infection and maintained during the 2 h viral attachment process. Then, the virus–drug mixture was replaced with fresh culture medium without drugs until the end of the experiment. No significant effect of pantethine was observed on the infection rate when the treatment was limited to 1 h before virus infection (Supplementary Fig. S4). A significant effect of pantethine (50–1000 µM) was observed when the pretreatment was prolonged to 24 h (Fig. 3A–D and Supplementary Fig. S6D), with a calculated IC50 of 28.84 µM (Fig. 3E). These results suggest an effect of pantethine on viral entry pathways.

### Pantethine reduced SARS-CoV-2 infection in Calu-3a cell cultures

Due to reported major differences in drug sensitivity and the viral entry pathways used by SARS-CoV-2 in in vitro models^31–35^, we also evaluated the efficacy of pantethine as an antiviral treatment in a different model using Calu-3a cells. In addition, Vero E6 cells cannot produce type I interferon, whereas Calu-3 cells are known to be interferon competent^33^. When comparing the 3 study conditions in the Vero E6 model, the effects of viral inhibition were highly significant in the post-entry and full treatment conditions. For the experiments on Calu-3a cells, we chose to continue our studies with only the full treatment condition, where the virus and drugs remain in the medium and in direct contact with the cells, mimicking the situation in the lungs during SARS-CoV-2 infection.

**Figure 4:**
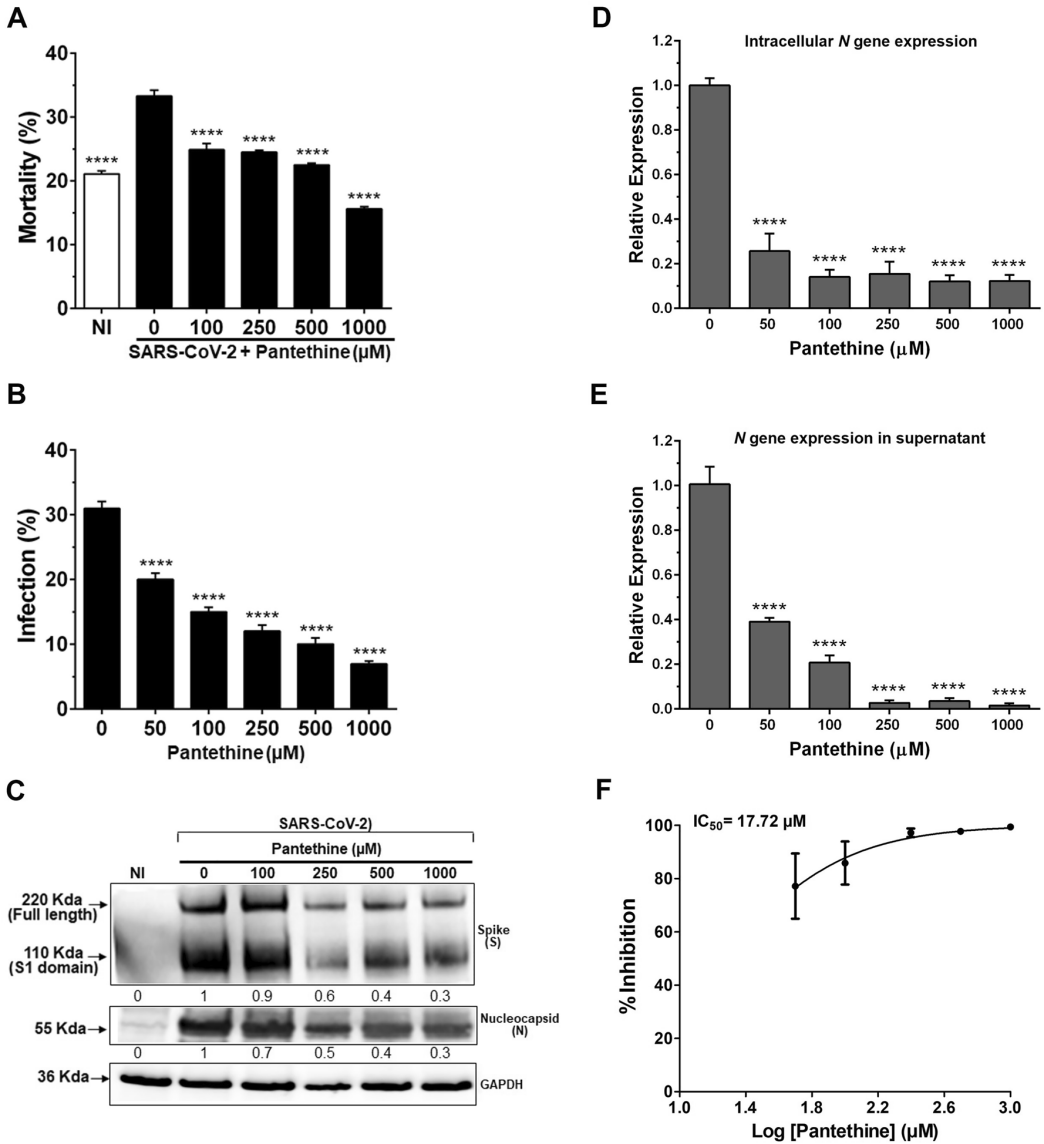
Pantethine treatment reduced SARS-CoV-2 infection in Calu-3 cell cultures. Full-time treatment with pantethine reduced the infection of Calu-3 cells by SARS-CoV-2 (MOI 0.05) significantly and in a dose-dependent manner. (**A**) Forty-eight hours post-infection, cells were collected and stained to determine viability and infection rates through the detection of the viral spike (S) protein in cells by flow cytometry analysis. Data represent the percentage of mortality and (**B**) the percentage of infection observed with the flow cytometry analysis experiments and are shown as the mean + SEM of results obtained from 3 independent experiments with 3 independent points per condition. (**C**) Forty-eight hours post-infection, cells were lysed with RIPA buffer, and western blot analyses were performed to detect the expression of the viral spike (S), the full length and S1 domain, and the nucleocapsid (N) proteins. GAPDH was used as a loading control. The numbers below the blots represent relative expression levels. For each viral protein, the levels of the uninfected cells were set at 1. Virus yields in infected cells (**D**) and in their supernatants (**E**) were quantified by qRT-PCR for the viral *N* gene. Calculated Ct values were converted to the fold-reduction of samples compared to the housekeeping gene *GAPDH* (for cells) or to noninfected cells (for supernatants) using the ΔΔCt method (fold change in viral RNA = 2^−ΔΔCt^). In (**D**) and (**E**), the results represent the mean + SEM. (**F**) An inhibitory dose–response curve based on viral *N* gene expression in supernatants was used to determine the IC50 using GraphPad Prism software. The results represent the mean ± SEM. In all experiments, the results were obtained from 3 independent experiments. ****p < 0.0001 compared to the control group (infected-untreated cells) by one-way ANOVA followed by Dunnett's post hoc test.

The efficacy of pantethine as an antiviral treatment was studied in Calu-3 cells infected with SARS-CoV-2 with a “full-time” treatment regimen. Treatment with pantethine reduced expression of the viral S and N proteins in Calu-3a cells (Fig. 4A,B). These observations were confirmed by significant reductions in viral S and N protein expression (Fig. 4C) and *N* and *NSP6* gene expression within cells (Fig. 4D and Supplementary Fig. S6E) and in the supernatant in cells treated with pantethine (Fig. 4E and Supplementary Fig. S6F), with a calculated IC50 of 17.72 µM (Fig. 4F). This reduction in infection after pantethine treatment was comparable to that achieved with remdesivir treatment (Supplementary Fig. S7B).

### Pantethine reduced intracellular cholesterol levels in non-infected and SARS-CoV2 infected Vero E6 cells

Total intracellular cholesterol levels were measured in non-infected Vero E6 cells treated with different concentrations of pantethine (250, 500 and 1000 µM) for 24 h (Short time incubation) and full treatment regimen for 72 h (Long time incubation). Lipids extraction was assessed at 72 h after treatment. Pantethine reduced total cholesterol levels in treated cells by about 35% after 24 h of treatment (Short time incubation) (Supplementary Fig. S8Aa and Ab), and by about 80% in the full treatment regimen where pantethine was added each day in the cell culture (Long time incubation) (Supplementary Fig. S8Ac and Ad). Similar results were obtained for the 3 different concentrations of pantethine (250, 500 and 1000 µM).

Due to these observed effects of the short and long time incubation with pantethine and the greater effect on cholesterol levels obtained with the long time incubation, we analyzed the total intracellular cholesterol levels in infected-SARS-CoV2 Vero E6 cells treated or not with pantethine according to the full treatment regimen for 72 h (Long time incubation). In our model, the virus reduced total cholesterol levels in infected Vero E6 cells. However, this reduction induced by the virus was not enhanced in infected cells treated with pantethine (Supplementary Fig. S8B).

In conclusion, our experiments demonstrate that pantethine reduces cellular cholesterol levels in non-infected cells, and that the virus also reduces total cholesterol levels in infected Vero E6 cells which might be due to the use of cholesterol by the virus. Therefore, one possible effects of pantethine on SARS-CoV-2 might be to alter cholesterol availability to the virus, among other protective mechanisms.

### Pantethine reduced the increase in HECT E3 ligase expression induced by SARS-CoV-2

Inhibition of HECT E3 ligases was proposed as a potential therapy for COVID-19^36^. We examined the expression of five members of this family in Vero E6 and Calu-3a cells by RT-PCR using the full-time treatment regimen for the same reasons explained in the above paragraph.

**Figure 5:**
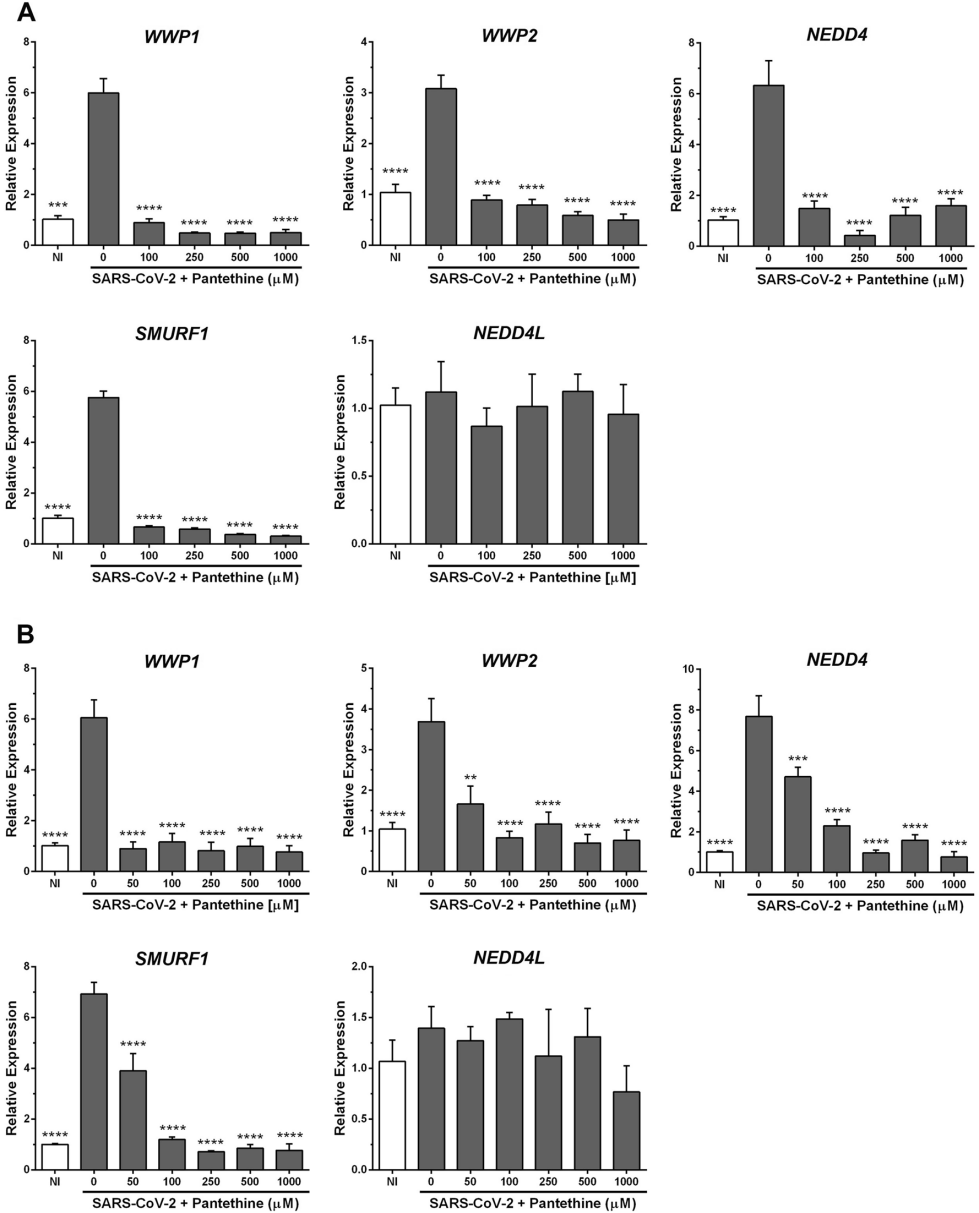
Pantethine reduced the increased HECT E3 ligase expression induced by SARS-CoV-2 infection in vitro. Vero E6 (**A**) or Calu-3 (**B**) cells infected with SARS-CoV-2 (MOI 0.05) were treated with pantethine (full-time treatment). Seventy-two (Vero E6) or forty-eight (Calu-3) hours post-infection, cells were collected, and their RNA was extracted to evaluate the expression levels of different HECT E3 ligases by qRT-PCR. Calculated Ct values were converted to the fold-reduction of samples compared to the housekeeping gene *GAPDH* using the ΔΔCt method (fold change in RNA = 2^−ΔΔCt^). The results represent the mean + SEM from 3 independent experiments. *p < 0.05, **p < 0.01 and ****p < 0.0001 compared to the control group (infected untreated cells) by one-way ANOVA followed by Dunnett's post hoc test.

As described previously^36^, SARS-CoV-2 infection significantly increased *WWP1*, *WWP2*, *SMURF1* and *NEDD4* mRNA expression and had no effects on *NEDD4-L* mRNA expression in Vero-E6 (Fig. 5A) and Calu-3a (Fig. 5B) cells. Pantethine (50–1000 µM) treatment inhibited the virus-induced increase in the mRNA expression levels of the investigated HECT E3 ligases, suggesting that the antiviral action of pantethine reported here could be mediated by a reduction in E3 ligase expression in Vero E6- and Calu-3a-infected cells. Pantethine treatment had no significant effect on noninfected cells (Supplementary Fig. S9).

### Pantethine had no effects on the virus-induced reduction in ACE2 mRNA expression

**Figure 6:**
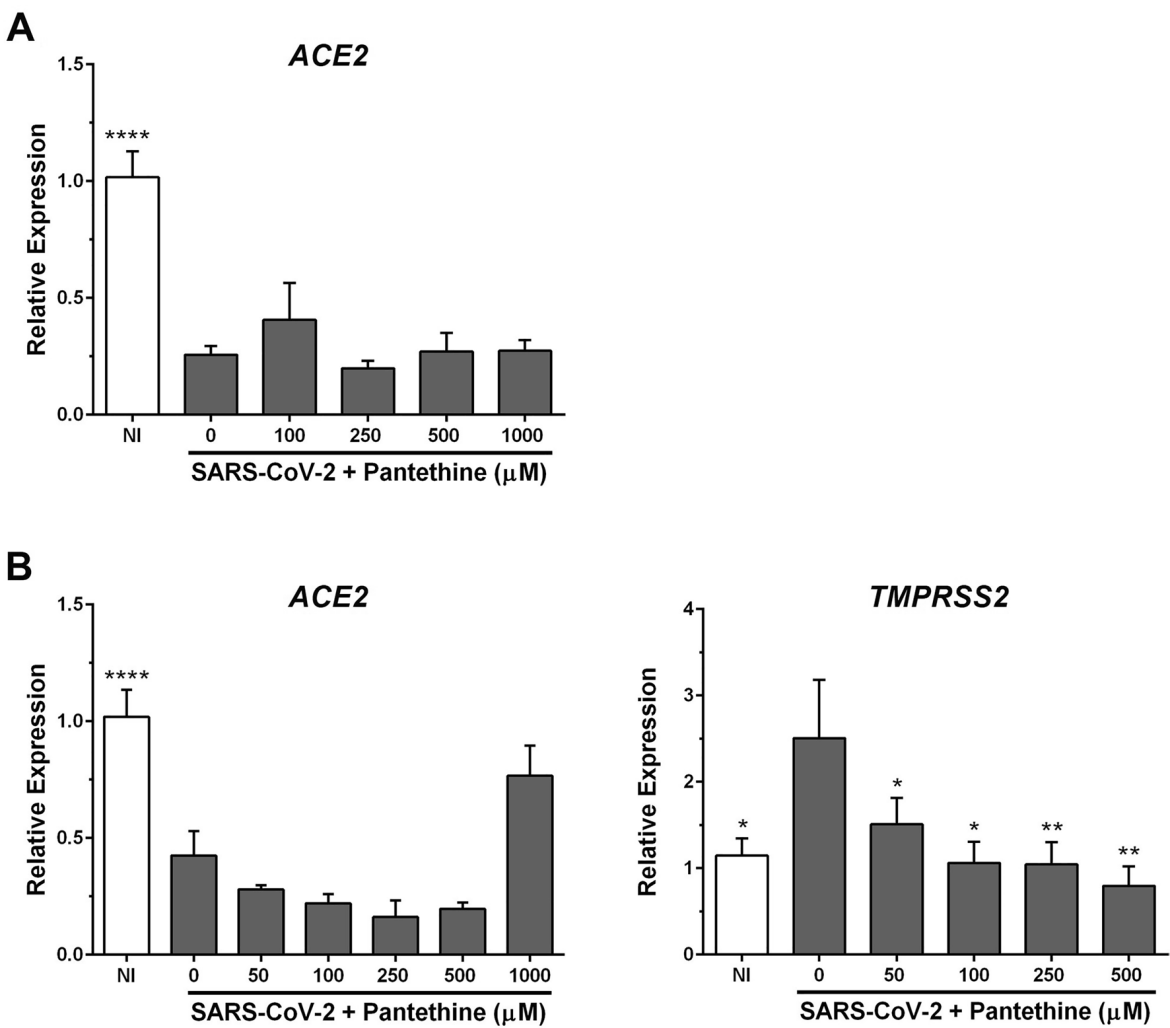
Effects of pantethine on ACE2 and TMPRSS2 expression in SARS-CoV-2-infected cells. Vero E6 **(A**) and Calu-3a (**B**) cells infected with SARS-CoV-2 (MOI 0.05) were treated with pantethine (full-time treatment). Seventy-two (Vero E6) or forty-eight (Calu-3) hours post-infection, cells were collected, and their RNA was extracted to evaluate the expression levels of *ACE2* and *TMPRSS2* (only for Calu-3a) by qRT-PCR. Calculated Ct values were converted to the fold-reduction of samples compared to the housekeeping gene *GAPDH* using the ΔΔCt method (fold change in RNA = 2^−ΔΔCt^). The results represent the mean + SEM from 3 independent experiments. *p < 0.05, **p < 0.01, ***p < 0.001 compared to the control group (infected untreated cells) by one-way ANOVA followed by Dunnett's post hoc test.

ACE2 was reported to be the main receptor used by SARS-CoV-2 to infect cells. As reported previously^37^, SARS-CoV-2 significantly reduced *ACE2* mRNA expression in infected Vero E6 (Fig. 6A) and Calu-3a cells (Fig. 6B). Pantethine treatment had no effect on this decrease in *ACE2* mRNA expression in infected cells (Fig. 6).

### Pantethine inhibited the increase in TMPRSS2 expression in infected Calu-3a cells

The docking of the SARS-CoV-2 S protein to the host ACE2 receptor is dependent on host proteases, including Transmembrane protease serine 2 (TMPRSS2). Targeting the expression levels of TMPRSS2 was proposed to be a rational approach to treat COVID-19^38^. In our experiments, SARS-CoV-2 increased *TMPRSS2* mRNA expression in Calu-3a cells (Fig. 6B). Pantethine treatments significantly inhibited the virus-induced increase in *TMPRSS2* mRNA expression in infected cells (Fig. 6B). These results suggest a potential effect of pantethine on virus entry into permissive cells through a decrease in *TMPRSS2* expression levels. Pantethine treatment had no significant effects on *TMPRSS2* expression in noninfected Calu-3a cells (Supplementary Fig. S10B).

### Pantethine inhibited the infection-induced increases in Interferonβ, TNFα, and IL6 expression in Calu-3a cells

Calu-3a are immunocompetent cells capable of sensing SARS-CoV-2 and secreting type I interferon and different inflammatory cytokines to protect against viral infection^31,37–40^. While the SARS-CoV-2-induced production of IFNβ occurs mainly through mitochondrial antiviral-signaling protein (MAVS) and interferon regulatory factor 3 (IRF3)^40,41^, that of IL6 and TNFα occurs through stimulator of interferon genes (STING), which activates nuclear factor kB (NF-κB)^42^.

**Figure 7:**
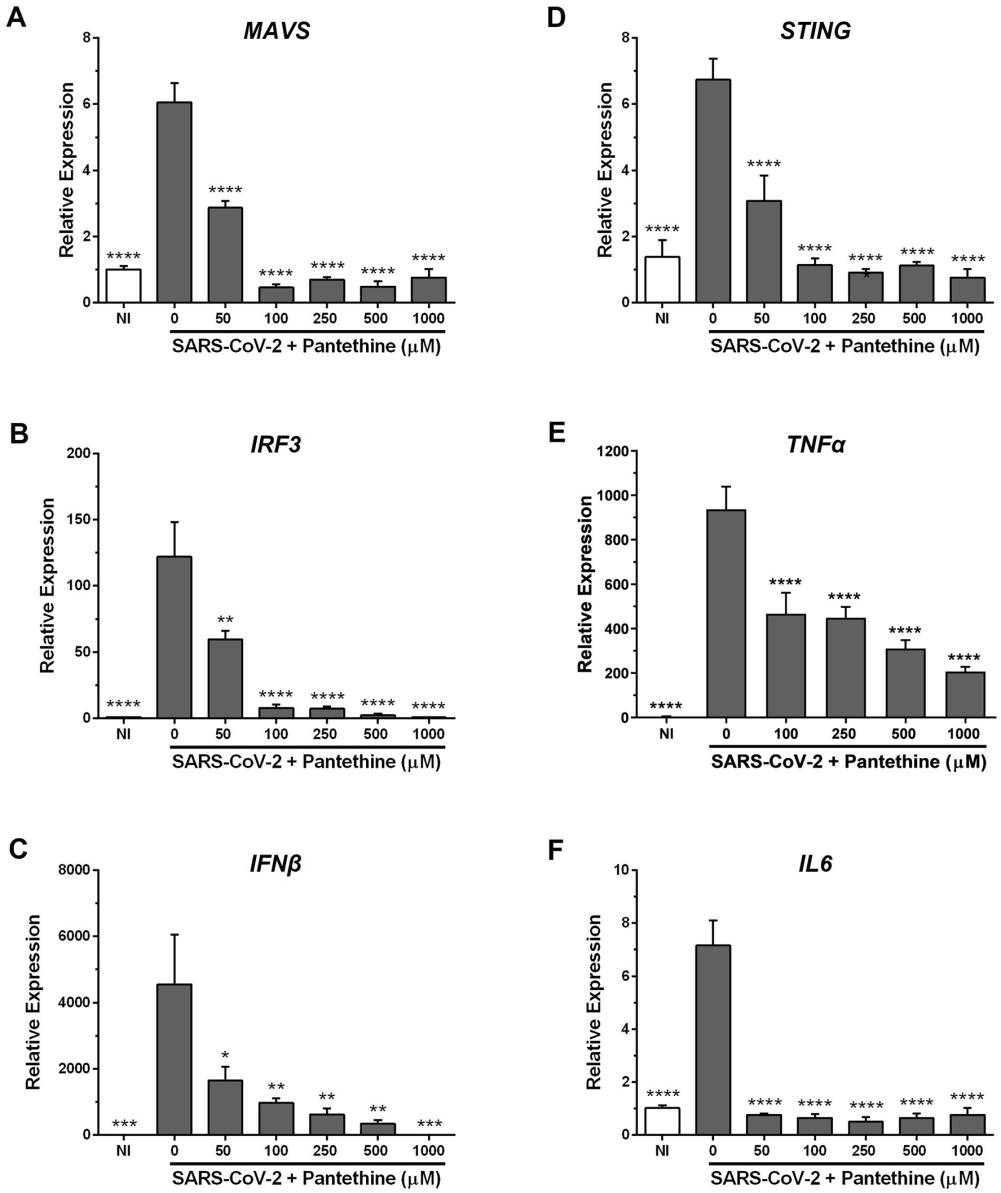
Pantethine reduced the inflammatory response induced by SARS-CoV-2 in Calu-3 cells. Calu-3 cells infected with SARS-CoV-2 (MOI 0.05) were treated with pantethine (full-time treatment). Forty-eight hours post-infection, cells were collected, and their RNA was extracted to evaluate the expression levels of different inflammatory genes by qRT-PCR. Calculated Ct values were converted to the fold-reduction of samples compared to the housekeeping gene *GAPDH* using the ΔΔCt method (fold change in RNA = 2^−ΔΔCt^). The results represent the mean + SEM from 3 independent experiments. **p < 0.01, and ****p < 0.0001 compared to the control group (infected-untreated cells) by one-way ANOVA followed by Dunnett's post hoc test.

In our experiments, SARS-CoV-2 infection significantly increased the expression of MAVS, IRF3 and IFNβ (Fig. 7A–C), as well as that of STING, TNFα and IL6 (Fig. 7D–F). Pantethine treatment (50–1000 µM) significantly reduced the mRNA expression of these molecules to levels comparable to those observed in noninfected cells (Fig. 7).

## Discussion

The low-molecular-weight thiol pantethine was reported to exert several physiological effects that could be involved in the SARS-CoV-2 pathogenic process. Here, we found that it significantly controlled SARS-CoV-2 infection in two in vitro experimental models, in Vero E6 cells and in Calu3a cells. This effect was demonstrated by a significant reduction in viral protein expression and attenuation of the infection-induced increases in the expression of *TMPRSS2*, HECT E3 ligases, genes associated with the interferon-β response and the inflammatory cytokines *TNFα* and *IL6*. Our results suggest that pantethine could exert its effects on both the entry and postentry SARS-CoV-2 pathogenic pathways with greater efficiency in the post-entry condition. In this work, we also described a simple flow cytometry method to estimate viability and the number of virus-infected cells in in vitro models of SARS-CoV-2 infection. This method could be developed further as a quick tool to screen the antiviral properties of potential molecules against different viruses.

One of the proposed strategies to treat COVID-19 is to prevent virus entry into host cells. The early steps of the process are initiated by the binding of the viral S protein to angiotensin-converting enzyme 2 (ACE2), which is localized in lipid raft domains^7,10,11^.

Rafts are cellular microdomains that are rich in saturated phospholipid chains tightly packed with cholesterol. Therefore, cholesterol is involved in the regulation of virus entry and membrane fusion^14,43–45^. A recent study reported that SARS-CoV-2 needs cholesterol to invade host cells and form mega cells^9^. Conversely, cholesterol depletion from cell membranes significantly reduced the infectivity of SARS-CoV^7,14^, which has pathogenic pathways similar to those of SARS-CoV-2^15^. In the present study, we show that pantethine, which is known to decrease the cholesterol content of cell membrane rafts and to alter their lipid composition^6^, exerted an antiviral effect.

Accordingly, in COVID-19 patients, statin therapy is associated with a better clinical outcome^46–48^. Similar to pantethine, statins inhibit 3-hydroxy-3-methyl-glutaryl-coenzyme A (HMG-CoA) reductase and thereby de novo cholesterol synthesis^49^. A recent study showed that SARS-CoV-2 infection reduced high-density lipoprotein (HDL) levels in patients, which increased progressively after successful treatment^50,51^. Here, long-term pantethine treatment was also reported to increase HDL serum levels^4^. Thus, one of the possible modes of action of pantethine could be a change in lipid raft composition, which was reported to induce ACE2 sequestration, preventing the correct exposure of ACE2 and thus altering the docking of the viral S protein^7,10^.

Furthermore, the docking of the viral S protein to host ACE2 is primed by different proteases, including the transmembrane protease serine 2 (TMPRSS2), which enables host cell fusion of the virus^11^. Reducing or inhibiting TMPRSS2 was proposed to reduce the infection rate and case severity by altering virus entry^11,51–55^. A recent paper reported that higher TMPRSS2/ACE2 ratios were associated with a prominent risk of severe COVID-19^56^. In our work, SARS-CoV-2 significantly reduced *ACE2* and increased *TMPRSS2* expression in infected cells, thus increasing the TMPRSS2/ACE2 ratio. Pantethine treatment inhibited the infection-induced increase in *TMPRSS2* expression in Calu-3a cells, suggesting that pantethine may affect SARS-CoV-2 entry by reducing the expression of *TMPRSS2*.

Our results show that pantethine have a significant antiviral effect when incubated with the cells 24 h prior to viral infection. On the contrary, no antiviral effect was obtained after 1 h of incubating pantethine with the cells. These results suggest that pantethine treatment may alter the lipid composition of cell membranes, which was reported before to decrease the cholesterol content and to alter the lipid composition of cell membrane rafts in human leukemic T cell line cultures^6^. Indeed, our experiments demonstrate that pantethine reduces cellular cholesterol levels in non-infected Vero E6 cells under a short time treatment regimen (Supplementary Fig. S8A), which might explain, at least in part, the reduction of SARS-CoV-2 infection observed in our “Entry” experiments where pantethine treatment was limited to 24 h before the virus addition to cultures (Fig. 3). In addition, when analyzing the full-treatment condition (Fig. 1), and the post-entry condition (Fig. 1) where pantethine was added each day to cultures (Long time incubation), a very high efficiency of pantethine was observed on the viral infection even at very low concentrations. These observations might also be explained, at least in part, by the important reduction of total cholesterol levels observed in non-infected cells treated with pantethine under the long time incubation regimen (Supplementary Fig. S8A).

Different papers reported that the virus is using the cholesterol to invade the cell. SARS-CoV-2 infection was also reported to cause lower serum cholesterol levels in COVID-19 patients (reviewed in^57^). Our experiments demonstrate that pantethine reduces cellular cholesterol levels. Therefore, one possible effect of pantethine on SARS-CoV-2 might be to alter cholesterol availability to the virus, among other protective mechanisms shown in our report.

HECT E3 ubiquitin ligases have been implicated in the cell egression phase of some RNA viruses^58–60^, including Ebola virus^61,62^. Recent studies have shown that the E3 ligase family members WWP1, WWP2 and NEDD4 are overexpressed in SARS-CoV-2-infected lung tissues in both mice and humans. Additionally, it has been demonstrated that NEDD4 and WWP1 interact and ubiquitylate the SARS-CoV-2 S protein and that blocking these molecules has an important antiviral effect by inhibiting viral egression, suggesting direct involvement of these proteins in the virus replication cycle^36^, as shown for other RNA viruses^58–62^. Another recent work reported that E3 ligase activity was critical for SARS-CoV-2 assembly and release through ubiquitination of the viral M protein, which was important for the interaction of the viral M and E proteins and the induction of autophagy^63^. Here, we also observed substantial increases in the mRNA expression of different HECT E3 ligases, specifically *WWP1*, *WWP2*, *SMURF1* and *NEDD4,* in infected Vero E6 and Calu-3a cells, which could exacerbate infection. Pantethine treatment inhibited this virus-induced increase in the mRNA expression levels of the investigated HECT E3 ligases, suggesting that the antiviral activity of pantethine could also occur through a reduction in viral egress. It is important to note that pantethine had no effect on the expression levels of E3 ligases in noninfected cells; thus, pantethine treatment does not seem to alter the expression of E3 ligases, whose activities are important for normal cellular functions, under normal conditions.

It is important to note that while pantethine was able to reduce the increase of TMPRSS2 and HECT E3 ligases expressions in infected cells, it had no effect on non-infected cultures. These observations suggest a direct or indirect mechanism linked to infection. Thus, additional investigation is needed to verify these observed effects of pantethine on TMPRSS2 and E3 ligases expression and how they interact with lipids.

An exaggerated immune response is one of the key features of COVID-19, mainly in patients with severe disease. In our Calu-3a model, in line with other studies, we observed increases in *IFNβ*, *TNFα*, and *IL6* expression in cells infected with SARS-CoV-2^39,42^. Pantethine treatment significantly reduced this infection-induced increase in *IFNβ* expression. Although IFNβ plays an essential antiviral role in COVID-19 by limiting SARS-CoV-2 propagation, a sustained increase in its expression is associated with aberrant inflammation and poor clinical outcomes^39^. This increase in *IFNβ* expression could be explained by the observed infection-induced increases in the expression of *MAVS* and *IRF3*, which were reported to be involved in IFNβ production^40,41^. Pantethine inhibited this increase in *MAVS* and *IRF3* expression in infected cells, as well as that of *STING,* which controls aberrant type I IFN production and cell death in COVID-19 through c-GAS-STING signaling^64^. In addition, the cGAS-STING pathway was reported to induce TNFα and IL6 production through NFkB^42^. Overall, the ability of pantethine to reduce the infection-induced increases in *MAVS*, *IRF3* and *STING* expression could explain the antiviral and anti-inflammatory effects of pantethine, which significantly inhibited *IFNβ*, *TNFα* and *IL6* expression in infected Calu-3a cells. Indeed, the inhibition of STING in mice reduced severe lung inflammation induced by SARS-CoV-2 and improved disease outcomes^39^.

Long COVID syndrome is defined as the persistence of COVID-19 symptoms for a long period after the initial infection. A clear understanding of its symptoms and pathophysiology is still lacking, but abnormal cholesterol levels, chronic inflammation, and severe COVID-19 symptoms, such as cardiovascular or neurological manifestations, are thought to be risk factors associated with long COVID^16,20,65,66^. In addition, recent studies proposed that one significant symptom of long COVID, intolerance to exercise, could be related to the formation of amyloid fibrin microclots in the blood, which induce poor oxygen delivery to different tissues^67,68^. Due to its different reported effects, pantethine might be used to prevent COVID-19, specifically severe cases and long COVID syndrome. Pantethine might attenuate the exacerbated immune response, and its hypolipidemic effect could be used in preventing the cardiovascular complications of COVID-19, as dyslipidemia is associated with increased mortality and severity of COVID-19^4,13–15,64^; pantethine could also be used to reduce microclot formation in the blood due to its reported anticoagulant effects^4,29^.

The most frequently applied dose of pantethine in human was 900 mg/day (3 × 300 mg/day), with a range of 600–1200 mg/day. The doses used in our study have been selected in the perspective of pantethine use in humans. The relative compound cystamine has similar effects at much lower doses, however, it is highly toxic and therefore cannot be used in patients, unlike pantethine. In humans, pantethine has been administered at various doses, such as 600–900 mg per day for 16 weeks^4^ with no adverse effects. Only moderate adverse effects, such as osmotic diarrhea, have been observed at the very high dose of 350 mg/kg/day^69^. Under our experimental conditions, we used high doses of pantethine; however, in view of a potential clinical application, an appropriate delivery device may improve the efficiency of treatment and allow a drastic reduction of the dose to be administered.

In conclusion, we described here, for the first time, the antiviral activity of pantethine, which might result from multiple convergent mechanisms during the entry-fusion phase, such as alteration of the composition and cholesterol content of lipid rafts and the cell membrane or inhibition of the infection-induced increase in TMPRSS2 expression. Pantethine could also exert its effects during subsequent stages of the SARS-CoV-2 replication cycle, for example, by disturbing intracellular mechanisms dependent on cholesterol or thiol-disulfide homeostasis or altering viral egression via inhibition of infection-induced HECT E3 ligase expression. Pantethine also presents additional nonlipid-related pleiotropic effects, such as anti-inflammatory, antioxidant, and anticoagulant effects^4,6,29,30,70^, that might confer benefits in patients with severe COVID-19 and long COVID syndrome. As it has already been used in humans with no significant adverse effects, pantethine displays a variety of mechanisms and functions that make it a potential tool for antiviral therapeutic intervention. Further investigations are required to validate this point using in vivo infection models.

## Methods

### Cells, viruses, and drugs

The Vero E6 African green monkey kidney cell line was kindly provided by Dr Andreola Marie-Aline, University of Bordeaux. Calu-3a cells were kindly provided by Dr Pierre Olivier Vidalain, CIRI Lyon UMR 1087.

Cells were maintained in Eagle’s medium (Dulbecco’s modified Eagle’s medium; Gibco Invitrogen supplemented with 10% heat inactivated FBS and 1% PS (penicillin 10,000 U/mL; streptomycin 10,000 µg/mL) (Gibco Invitrogen) at 37 °C in a humidified atmosphere of 5% CO_2_. For Calu-3a cells, Tryple Express was used (Gibco 12604013). The strain BetaCoV/France/IDF0372/2020 was supplied by the National Reference Centre for Respiratory Viruses hosted by Institut Pasteur (Paris, France) and headed by Pr. Sylvie van der Werf. The human sample from which strain BetaCoV/France/IDF0372/2020 was isolated was provided by Dr. X. Lescure and Pr. Y. Yazdanpanah from the Bichat Hospital, Paris, France. Moreover, the strain BetaCoV/France/IDF0372/2020 was supplied through the European Virus Archive goes Global (Evag) platform, a project that has received funding from the European Union’s Horizon 2020 research and innovation programme under grant agreement No. 653316. The virus titer used for all experiments was 3.75E + 6 PFU/mL. All infection experiments were performed in a biosafety level-3 (BLS-3) laboratory at either the CRC (Cordelier Research Center) or UB’L3 facility (TBMcore, Bordeaux). Pantethine was purchased from Sigma-Aldrich and Clinisciences (Cat no. HY-B1028), and remdesivir was purchased from COGER (Cat no. AG-CR1-3713-M005).

### Evaluation of antiviral activity, toxicity, and infection inhibition

To evaluate the toxicity of pantethine to Vero E6 cells and its antiviral efficacy, we measured the percentage of viable cells and the percentage of infected cells by flow cytometry.

Vero-E6 cells were cultured overnight in 24-well cell culture dishes at a density of 75 × 10^3^ cells/well, and Calu-3a cells were cultured 4 days before the experiments at a density of 1.50 × 10^6^ cells/well. The timing of the addition experiment is detailed in the next paragraph. Drugs were added to the cell culture each day at the same concentration. For Vero E6 cells, the time 72 h post-infection has been chosen after several developmental experiments (Supplementary Fig. S3) and the time 48 h post-infection for Calu-3a cells (Supplementary Fig. S5). The cell supernatant was collected and frozen immediately at − 80 °C for viral extraction and q-PCR amplification. The cells were collected, and a portion was analyzed by flow cytometry to measure the inhibition of infection by intracellular staining against Spike protein (SARS-CoV-2 Spike Protein-Alexa 647, Cat no. 51-6490-82, eBioscience) using a Cytofix/cytoperm fixation permeabilization kit (Cat no. 554714, BD) according to the manufacturer’s instructions. Toxicity was analyzed by using Viobility 405/452 Fixable Dye (Cat no. 130-109-814, from Miltenyi Biotec) according to the manufacturer’s instructions. Briefly, the cells were washed twice with PBS before viability fixable dye staining for 15 min at room temperature. Then, the cells were permeabilized with Cytofix/Cytoperm buffer for 20 min, and after two washes with Permash buffer, anti-spike-Alexa 647 was added to the cells for 30 min at 4 °C. After staining, the cells were fixed with 2% paraformaldehyde (FPA) and then analyzed on a Fortessa Flux Cytometer. Thirty thousand events were recorded for each condition in triplicate. Analyses were performed using Kaluza software (Beckman Coulter Life Sciences). The remaining cells were lysed either in RIPA lysis buffer (Invitrogen, Cat no. 10230544) containing protease (Roche) and phosphatase inhibitors (Invitrogen) for further quantification and immunoblotting analysis or in LBP buffer for RNA purification and RT–qPCR analysis. Each condition was performed in triplicate (n = 3) in each experiment, and each experiment was repeated 3 times independently.

### Pantethine treatment

Pantethine and remdesivir were used for the time-of-addition experiment. Vero E6 cells (75 × 10^3^ cells/well) were treated with pantethine or remdesivir at different stages of virus infection. For “full-time” treatment, cells were pretreated with the drugs for 1 h prior to virus infection, followed by incubation with the virus for 2 h in the presence of the drugs until the end of the experiment. For “entry” treatment, the drugs were added to the cells for 1 h or 24 h before virus infection and maintained during the 2-h viral attachment process. Then, the virus–drug mixture was replaced with fresh culture medium without drugs until the end of the experiment. For the “post-entry” experiment, virus was added to the cells to allow infection for 2 h, and then the virus-containing supernatant was replaced with drug-containing medium until the end of the experiment. For all the experimental groups, cells were infected with virus at a multiplicity of infection (MOI) of 0.05, and at 72 h post-infection, for Vero E6 cells and at 48 h post-infection for Calu-3a cells (Supplementary Figs. S3 and S5). Cell supernatant and cell lysates were collected for qRT-PCR and western blot analysis, respectively. Cells were also analyzed by flow cytometry to determine viability and viral replication levels by analyzing the intracellular expression of the spike protein.

### RNA extraction and quantitative real-time RT-PCR (qRT-PCR)

#### Viral RNA extraction from the supernatant

Two hundred microliters of cell culture supernatant were harvested for viral RNA extraction using the MiniBEST Viral RNA/DNA Extraction Kit (Takara, Cat no. 9766) according to the manufacturer’s instructions. RNA was eluted in 30 µL of RNase-free water.

#### Intracellular RNA purification

After being washed with PBS, the cells were lysed with LBP and stored at − 80 °C. ARN purification was performed using the “Nucleospin RNA PLUS” kit according to the manufacturer’s recommended procedures (Machery Nagel ref #740984.250).

#### Quantitative real-time RT-PCR (qRT-PCR)

Total RNA was converted to cDNA using the PrimeScript RT Reagent Kit with gDNA Eraser (Takara, Cat no. RR047A) following the manufacturer’s recommended procedures. Quantitative PCR was performed using TB Green Premix Ex Taq II (Takara Cat no. RR820A). Briefly, each reaction consisted of a total volume of 25 μL containing 1 μL of each primer [0.4 µM/μL], 2 μL of cDNA (5 ng/µL), 12.5 μL of TB Green Premix Ex Taq II and 8.5 μL of RNase-free water.

Real-time PCR was performed using a Bio Rad CFX384 Real-Time PCR Machine. The thermal cycling conditions used were as follows: initial denaturation at 95 °C for 30 s, followed by 40 cycles of amplification at 96 °C for 5 s and 60 °C for 30 s. The primers used for the SARS-CoV-2 N, NSP6 and spike genes were designed and purchased from Eurofins. The different primers used in qRT-PCR studies are described in Table 1.

**Table 1:** The primers used in the qRT-PCR studies.

N	Fw	CGTTTGGTGGACCCTCAGAT
	Rv	CCCCACTGCGTTCTCCATT
NSP6	Fw	GGTTGATACTAGTTTGTCTGGTTTT
	Rv	AACGAGTGTCAAGACATTCATAAG
GAPDH	Fw	AAGGTCGGAGTCAACGGATTT
	Rv	TGAAGGGGTCATTGATGGCA
WWWP1	Fw	TGTAAATGTTACGCCACAGACT
	Rv	GCTTGTTTCAAATCTATCGTTGC
WWWP2	Fw	GAAAGTGGTGTCCGCAAAGC
	Rv	ATGACTCTGTGCCGTGACATT
NEDD4	Fw	CTGCTACGGACAATTATACCCTA
	Rv	CATCCAACAGTTTGCCATGATA
NEDD4-L	Fw	ACGTAGCGGATGAGAATAGAGAAC
	Rv	CTGTGATTAGATGGGTTTACCCTGA
SMURF1	Fw	CCGCTCCAAGGCTTCAAGG
	Rv	ATCCGGTTAAAGCAGGTATGGG
ACE-2	Fw	GGGATCAGAGATCGGAAGAAGAAA
	Rv	AGGAGGTCTGAACATCATCAGTG
TMPRSS2	Fw	AATCGGTGTGTTCGCCTCTAC
	Rv	CGTAGTTCTCGTTCCAGTCGT
STING	Fw	TACATCGGATATCTGCGGCTG
	Rv	CGGTCTGCTGGGGCAGTTTATC
IFN-b	Fw	GGCACAACAGGTAGTAGGCG
	Rv	AAGCCTCCCATTCAATTGCC
IL-6	Fw	GAGAAAGGAGACATGTAACAAGAG
	Rv	CCTCTTTGCTGCTTTCACAC
MAVS	Fw	CCGAGTCTCGTITCCTCTC
	Rv	CTGAAATTGCGGCAGATATAC
IRF3	Fw	CTGATACCCAGGAAGACATTC
	Rv	GGGCCAACACCATGTTAC

SARS-CoV-2 cDNA (Ct ~ 20 for the N and NSP6 genes) was used as a positive control for viral gene expression in the supernatant.

Relative intracellular mRNA quantities were normalized to the GAPDH mRNA level, and the expression fold change was calculated using the ΔΔCt method. Each experiment was repeated 3 times.

### Western blot analysis

For western blot analysis, protein samples were separated by 4–12% NUPAGE SDS–PAGE (Invitrogen) and then transferred to nitrocellulose membranes (Amersham Bioscience). After blocking with 5% BSA in TBS buffer containing 0.05% Tween 20, the blot was probed with a mouse anti-spike antibody (S1-NTD) (E7M5X) (1:2000, Ozyme, Cat. No. 42172S) and an anti-N antibody (1:10,000 dilution, Fisher Scientific, Cat. No. MA536086) as primary antibodies and horseradish peroxidase (HRP)-conjugated goat anti-mouse IgG or goat anti-rabbit IgG (Invitrogen) as the secondary antibody. Protein bands were detected with ECL Chemiluminescent substrate (Pierce) using a CCD camera (Syngene Pxi-4).

### Immunocytochemistry and confocal microscopy

At the end of the incubation times, the cells were fixed with 4% PFA for 20 min at room temperature. Cells were washed in PBS and incubated for 30 min in PBS with 5% normal donkey serum (NDS, Sigma-Aldrich) and 0.1% Triton X-100. The primary antibody (1:1000; SARS-CoV/SARS-CoV-2 (COVID-19) spike antibody [1A9]; Gene Tex, CA 92606 USA) was diluted in PBS with 1% NDS and 0.1% Triton X-100 and incubated overnight at 4 °C. Cells were washed in PBS and subsequently incubated with a Alexa Fluor 488 (A488)-conjugated donkey anti-rabbit secondary antibody (1:300; Life Technologies, Molecular Probes) diluted in PBS with 1% NDS and 0.1% Triton X-100 for 1 h at RT. Cells were rinsed in PBS, stained with DAPI 1:1000 in PBS for 2 min at RT, rinsed and incubated with PBS for confocal microscopy analysis.

Cells were analyzed using a Leica TCS SP8 confocal scanning system (Leica Microsystems, Wetzlar, Germany) equipped with a 405‐nm diode, 488‐nm Ar, 561‐nm DPSS and 633‐nm HeNe lasers. Eight-bit digital images were collected from a single optical plane using a 40 × HC PL APO CS2 oil-immersion Leica objective (numerical aperture 1.30). For each optical section, double-fluorescence images were acquired in sequential mode to avoid potential contamination caused by crosstalk of linkage-specific fluorescence emissions. The settings for laser intensity, the beam expander, the pinhole (1 Airy unit), the range property of the emission window, electronic zoom, gain and offset of the photomultiplicator, field format, and scanning speed were optimized initially and held constant throughout the study so that all sections were digitized under the same conditions. Composite illustrations were generated in Adobe Photoshop CS3 (Adobe Systems, San Jose, CA, USA).

### Quantitation of intracellular cholesterol

Amplex Red cholesterol assay kit (Invitrogen, Thermo Fisher Scientific) was used to measure intracellular cholesterol as described in Ref.^71^ with minor modifications. Briefly, Vero-E6 cells were infected as described previously applying “Full treatment” regimen and “Entry” with 24H of treatment with pantethine before infection. After 72H cells were washed 3 times with cold PBS. For each well, 2 mL of hexane: isopropanol mixture at the ratio of 2:1 was added and incubated for 30 min at RT. After the incubation, the lipid containing supernatant was transferred to a glass tube and the organic solvent was evaporated using a gentle flow of nitrogen. The lipid was extracted again using chloroform and 1% Triton X-100 and dissolved in 1 × reaction buffer (provided in the kit). The subsequent procedures were carried out according to the instructions provided by the manufacturer. The cell skeleton left on the plate was dissolved in 200 µL of RIPA containing 1 × anti-proteases and anti-phosphatases. Afterwards, protein concentration was measured (ThermoFischer Scientific cat#23235) and used to standardize the cholesterol concentration for comparison between different groups.

### Statistical analysis

Statistical analysis of means was performed using *one-way ANOVA followed by Dunnett's post* hoc *test* to determine significance using GraphPad Prism software (GraphPad Software Inc., USA). Values are given as the mean + S.E.M. and a *p *value < 0.05% was considered to indicate significance.

## Supplementary Information

Supplementary Figures.

## Data Availability

The datasets used and/or analysed during the current study available from the corresponding author on reasonable request.
