# Supplementary material for: Potential antiviral effects of pantethine against SARS-CoV-2
Source: Sci Rep. 2023 Feb 8;13:2237. doi: 10.1038/s41598-023-29245-0 (PMC9906591; doi:10.1038/s41598-023-29245-0)
Supplement: Supplementary file 1 — Supplementary Figures. [file 41598_2023_29245_MOESM1_ESM.pdf]

Supplementary Figure S1

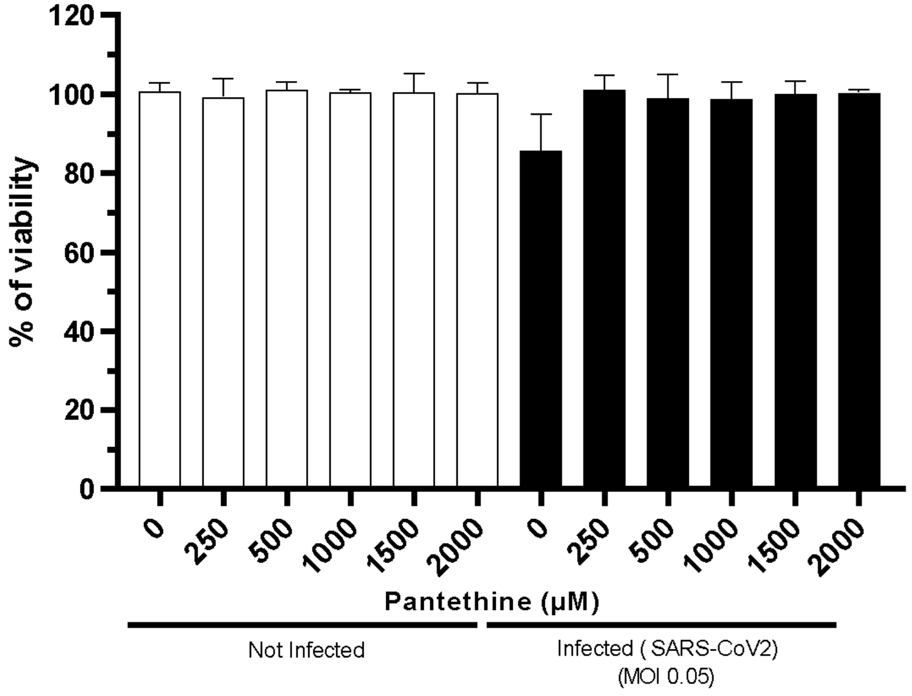

Supplementary Figure S1. Pantethine was not toxic on Vero E6 cells cultures.

Vero E6 cells, non-infected or infected with SARS-CoV-2 (MOI 0.05), were incubated with different concentrations of pantethine. Seventy-two hours post-infection, cells were collected, and the percentage of dead cells were evaluated by the Viability 405/452 Fixable Dye (Miltenyi Biotec) and detected using flow cytometry analysis. No toxicity was observed with the different doses of pantethine used for 72 h of treatment in all studied conditions.). Results represent mean + SD, obtained from 3 independent experiments.

Supplementary Figure S2

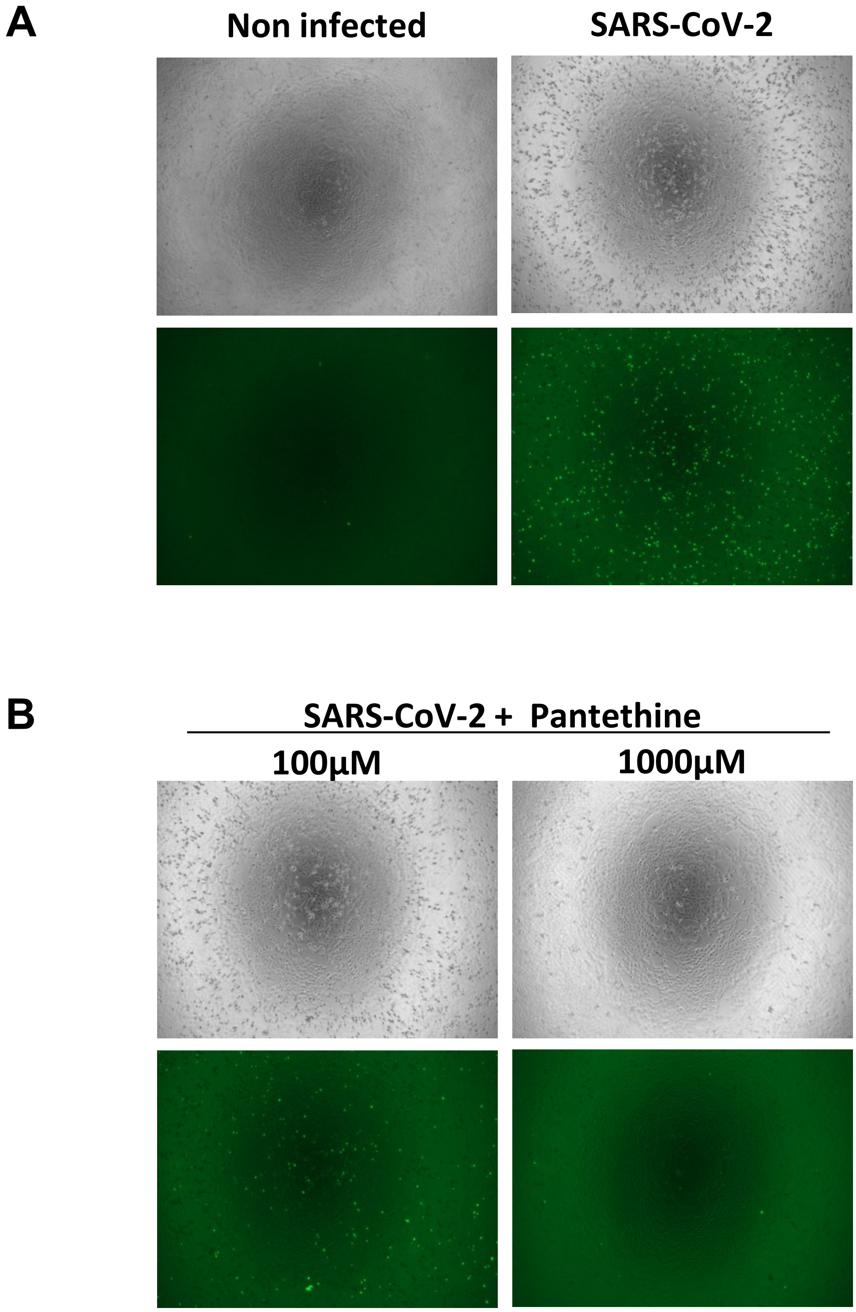

Supplementary Figure S2.Observation of cytopathic effect.

Vero E6 cells non infected or infected with SARS-CoV-2 at MOI 0.05 were incubated with the different concentration of pantethine. After 72 h pictures were taken using transmitted light on Evos microscope at a x4 magnification. Dead cells were stained using CellTox and observed under similar conditions using the GFP channel.

Supplementary Figure S3

The setup of SARS-CoV-2 infection in Vero E6 cell cultures

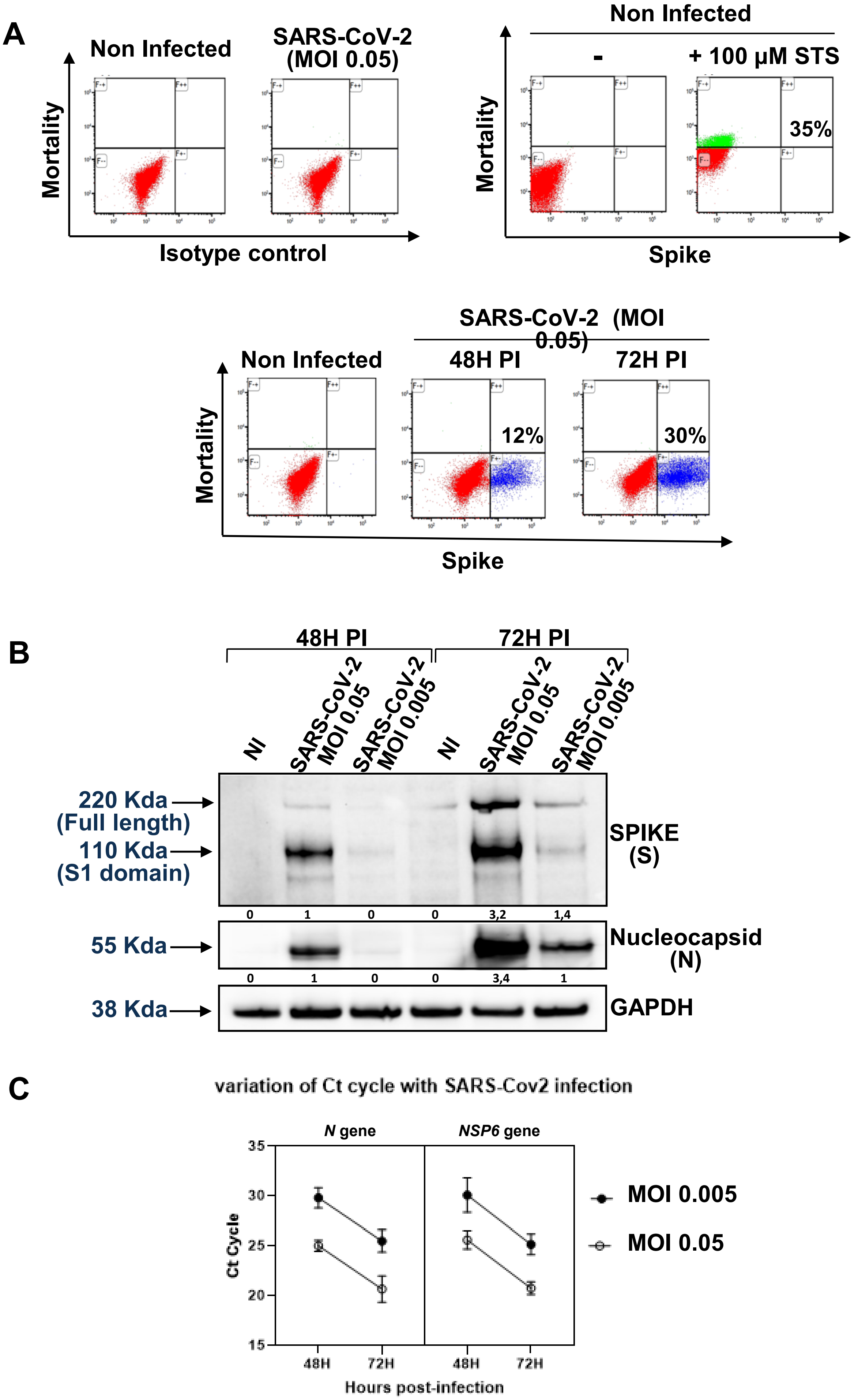

Supplementary Figure S3. The setup of infectious system with SARS-CoV-2 in Vero-E6 cells.

Cells were treated or not with staurosporine and infected with SARS-CoV-2 (MOI 0.05 or 0.005) **(A)** Forty-eight- or seventy-two hours post-infection, cells were collected and stained for mortality and infection rate through the detection of the viral Spike (S) protein in cells by flow cytometry analysis. The presented data, with the percentage of infection in each plot, are representative of 1 of 3 independent experiments that gave similar results. **(B)** Forty-eight- or seventy-two-hours post-infection, cells were lysed by RIPA buffer and western blot analyses were performed to detect the expression of the viral Spike (S), the full length and S1 domain, and the Nucleocapsid (N) proteins. GAPDH was used as loading control. The numbers below the blots represent relative expression levels. For each viral protein, levels of the infected-untreated cells were set at 1. **(C)** Virus yields in the supernatants were quantified by qRT-PCR for the viral *N* and *NSP6* gene. The presented Ct values represents the mean of 3 independents experiments that gave similar results.

Supplementary Figure S4

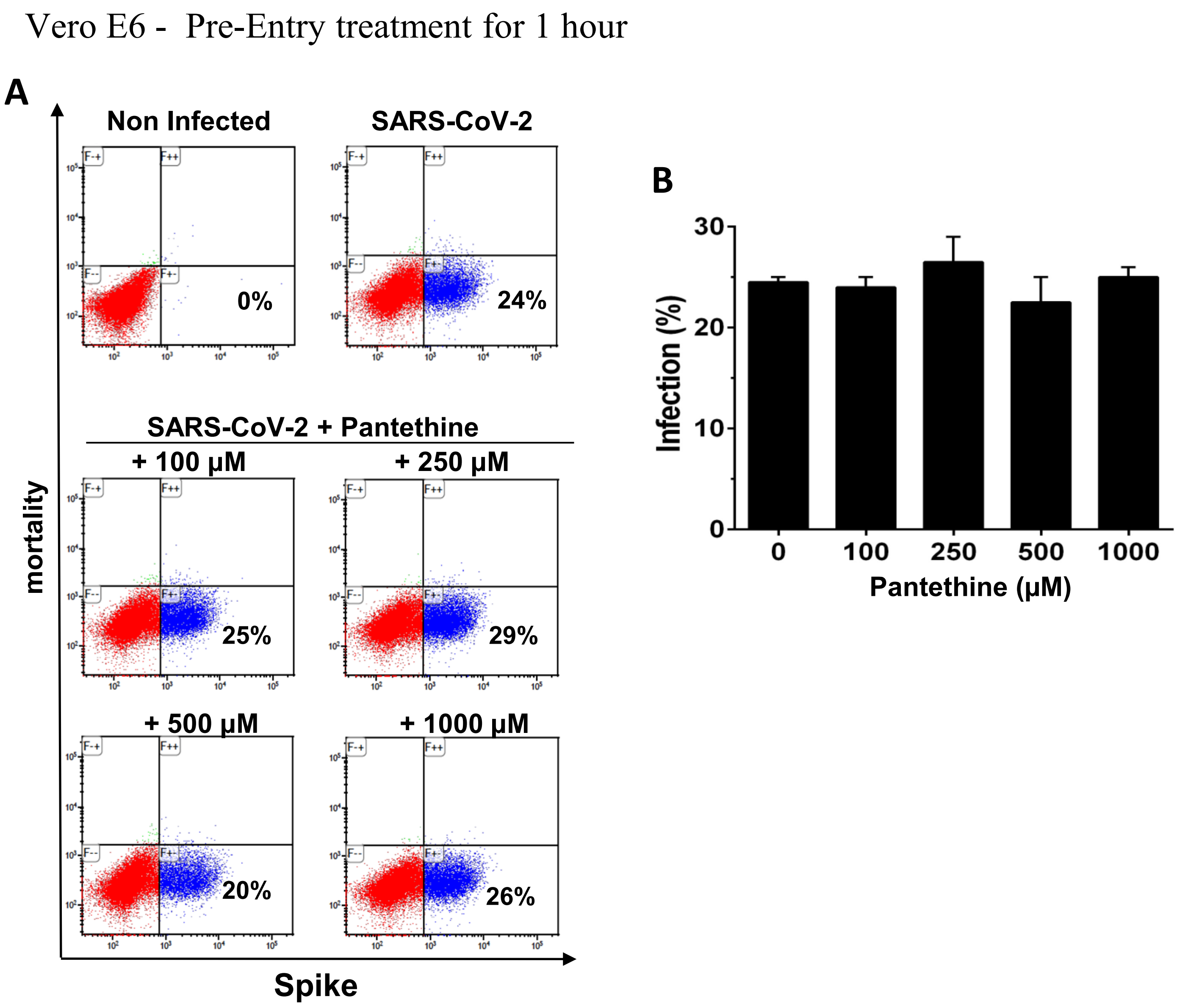

Supplementary Figure S4. Pantethine Pre-entry treatment for 1 hour before SARS-CoV-2 infection in Vero E6 cells.

Pantethine (100-1000µM) was added to cells for 1 hour before virus infection (MOI 0.05) and maintained during the 2 hours viral attachment process. Then, the virus-drug mixture was replaced with fresh culture medium without drugs till the end of the experiment. Seventy-two hours post-infection, cells were collected and stained for mortality and infection rate through the detection of the viral Spike (S) protein in Vero E6 cells by flow cytometry analysis. (**A**) The presented data, with the percentage of infection in each plot, are representative of 1 of 3 independent experiments that gave similar results. **(B)** Data represents the % of infection observed with the cytometry-analysis experiments and are shown as mean + SEM of results obtained from 3 independent experiments. No significant effect of pantethine was observed on the infection rate when the treatment was limited to 1 hour before virus infection.

Supplementary Figure S5

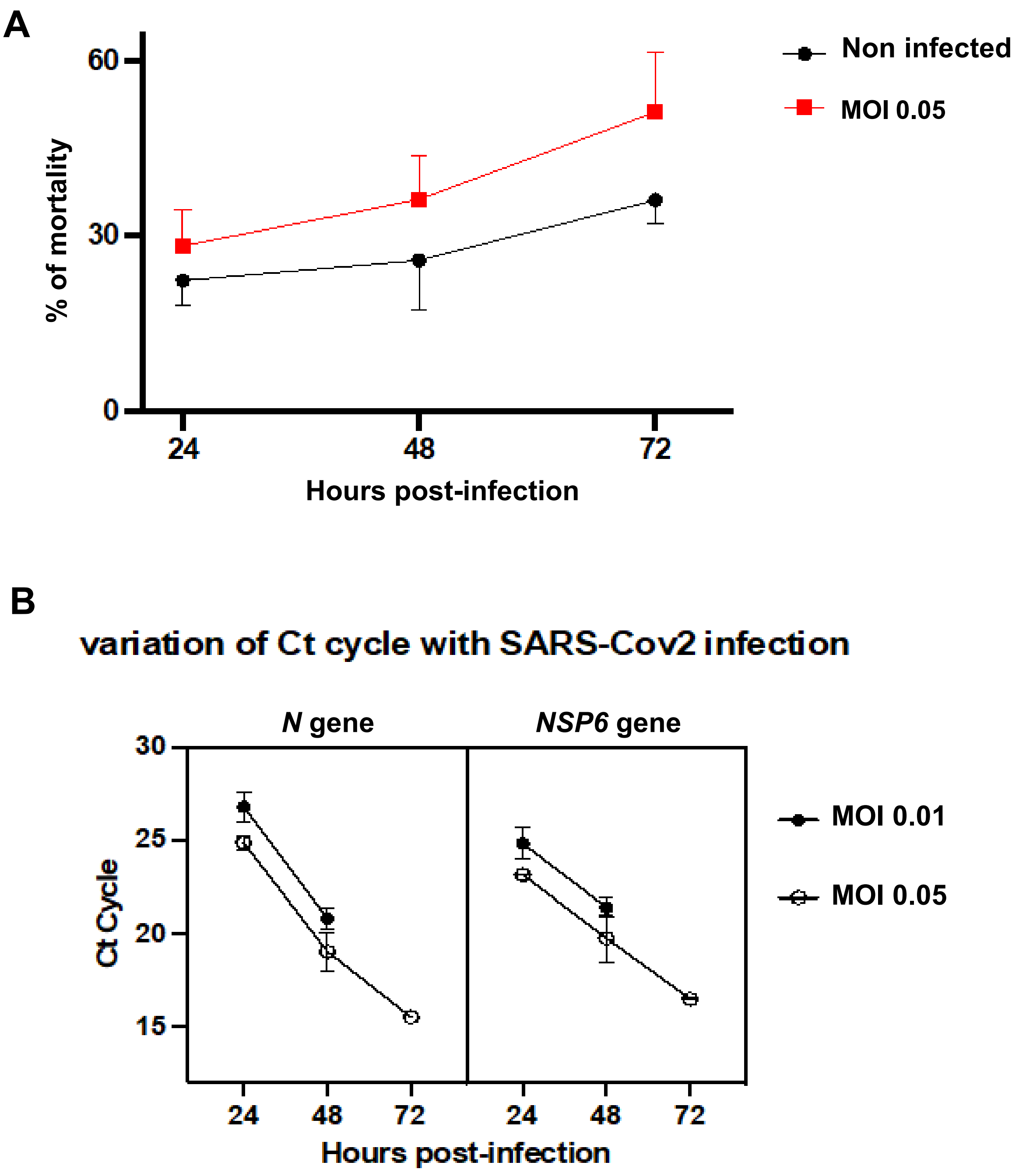

Supplementary Figure S5. The setup of infectious system with SARS-CoV-2 in Calu-3a cells.

Calu-3a cells were infected with SARS-CoV-2 (MOI 0.01 or 0.05). **(A)** Twenty-four, forty-eight- or seventy-two-hours post-infection, cells were collected and % of dead cells were detected using flow cytometry analysis. The presented percentages are representative of 1 of 3 independent experiments that gave similar results. **(B)** Twenty-four, forty-eight- or seventy-two-hours post-infection, Virus yields in the supernatants were quantified by qRT-PCR for the viral *N* and *NSP6* gene. The presented Ct values represents the mean of 3 independents experiments that gave similar results.

Supplementary Figure S6

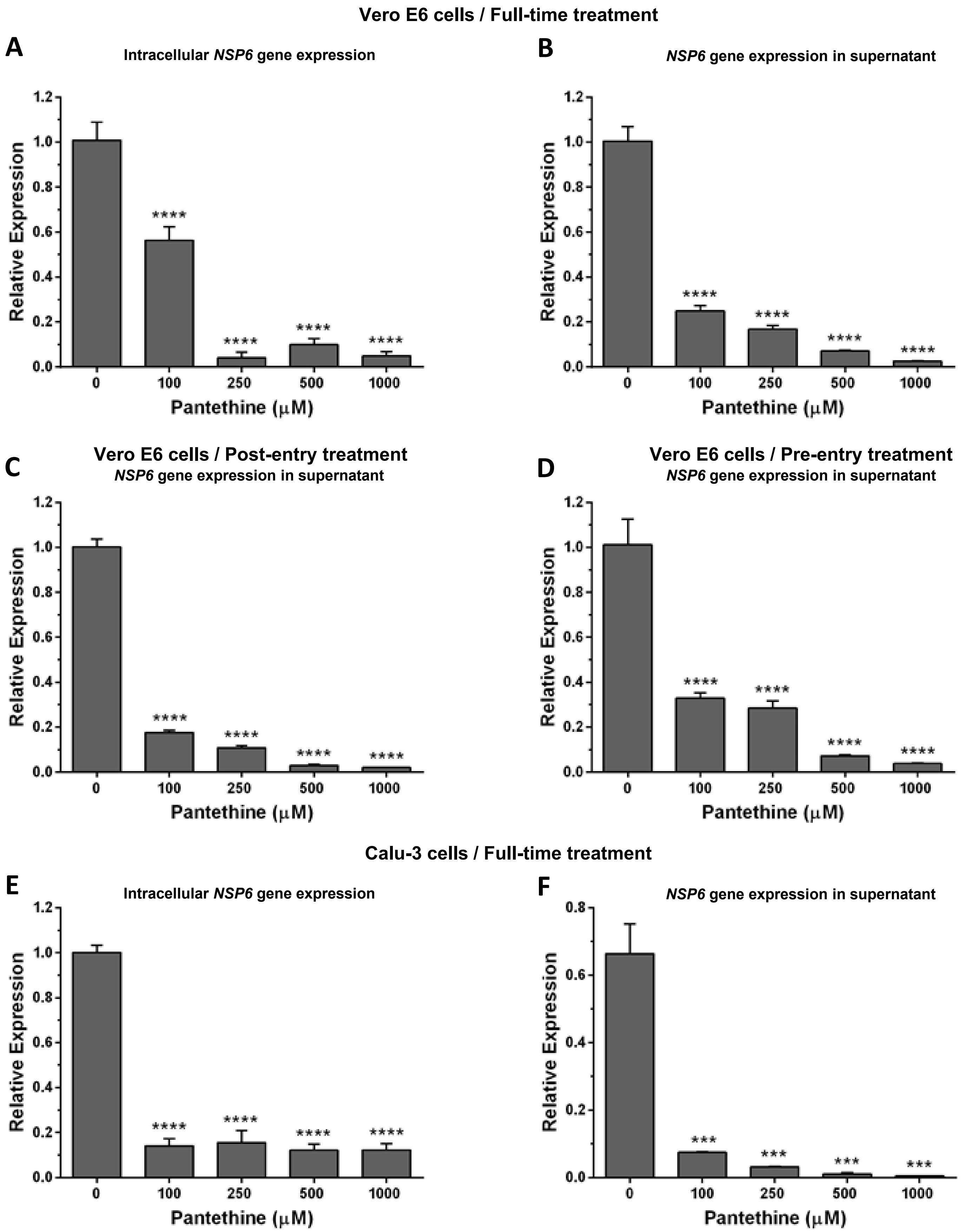

Supplementary Figure S6. Viral NSP6 gene expression in infected cultures treated or not with pantethine.

Vero E6 (**A – D**) and Calu-3a (**E-F**) cells were infected by SARS-CoV-2 (MOI 0.05) and treated or not with pantethine. For pantethine treatments, Full-time (**A, B, E, F**), Post-entry (**C**) or Pre-entry (**D**) regimens were used. Seventy-two hours (Vero E6) or forty-eight hours (Calu-3a) post-infection, cells were collected, their RNA extracted, and the viral *NSP6* gene expression was quantified by qRT-PCR in infected cells or their supernatants. Calculated Ct values were converted to fold-reduction of samples compared to the housekeeping gene *GAPDH* (for cells) or to the non-infected cells (for supernatants) using the ΔΔCt method (fold changed in viral RNA=$2^{-\Delta\Delta Ct}$). In B, E and F, results represent mean + SEM. Results were obtained from 3 independent experiments. ***$p < 0.001$ and ****$p < 0.0001$ compared to the control group (infected-untreated cells) by *One-way ANOVA test* followed by *Dunnett's post*-hoc *test*.

Supplementary Figure S7

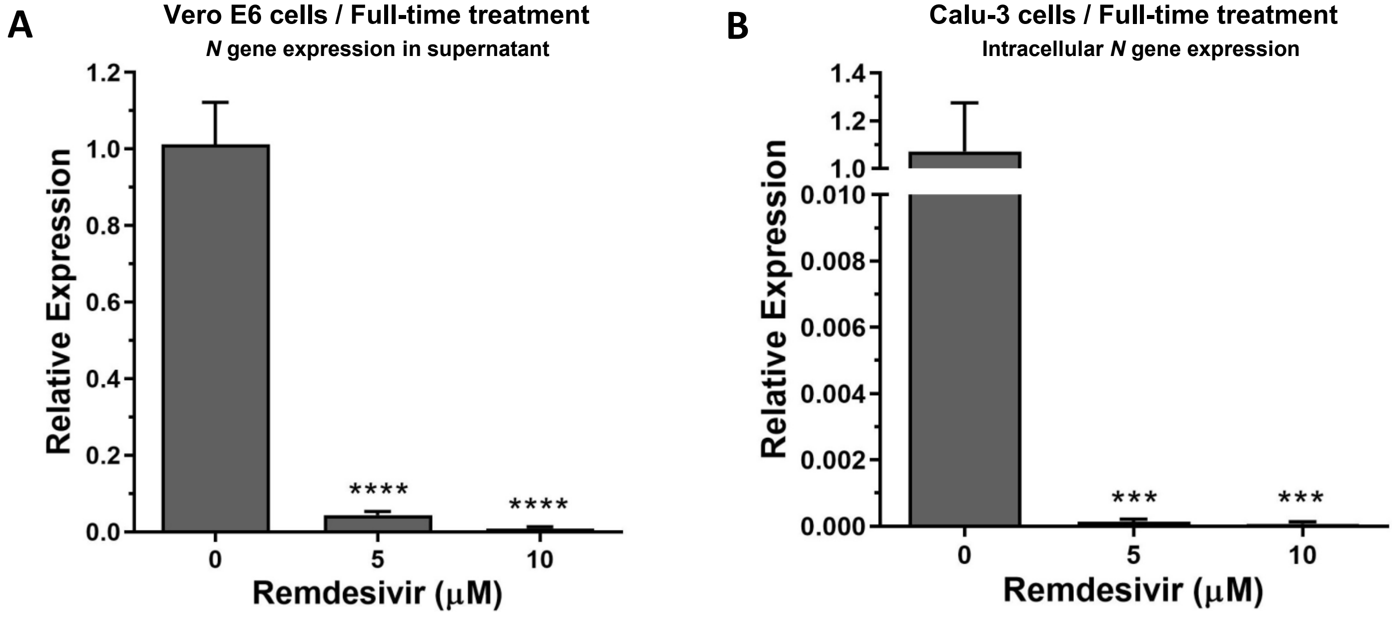

Supplementary Figure S7. Full-time treatment with remdesivir reduced the viral N gene expression in Vero-E6 and Calu-3a cells infected by SARS-CoV-2.

Vero E6 (**A**) and Calu-3a cells (**B**), infected with SARS-CoV-2 (MOI 0.05) were treated or not with remdesivir (Full-time treatment). Seventy-two hours (Vero E6) or forty-eight hours (Calu-3a) post-infection, cells were collected, their RNA extracted, and the viral *NSP6* gene expression was quantified by qRT-PCR in infected cells or their supernatants. Calculated Ct values were converted to fold-reduction of samples compared to the housekeeping gene *GAPDH* (for cells) or to the non-infected cells (for supernatants) using the ΔΔCt method (fold changed in viral RNA=2^-ΔΔCt). Results represent mean + SEM, obtained from 3 independent experiments. ***$p < 0.001$ and ****$p < 0.0001$ compared to the control group (infected-untreated cells) by *One-way ANOVA test* followed by *Dunnett's post*-hoc *test*.

Supplementary Figure S8

A Non-infected Vero E6 cells

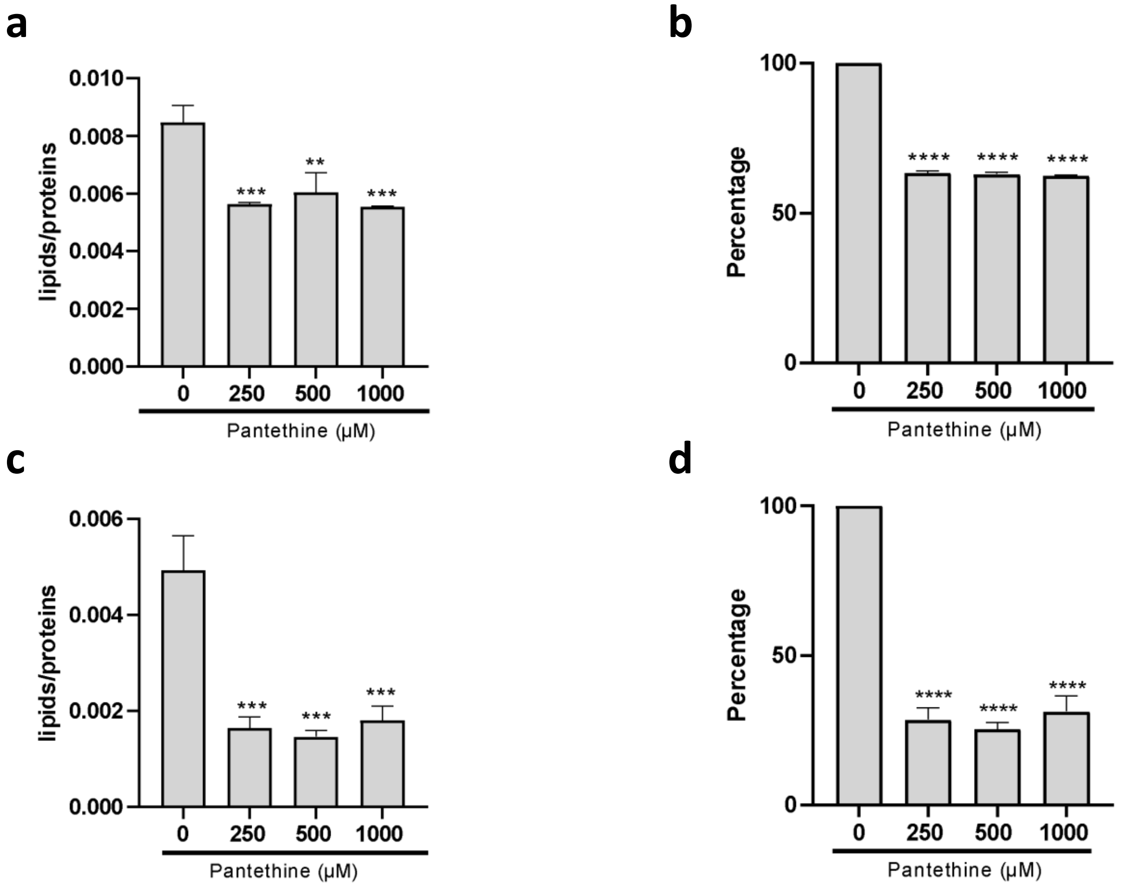

B SARS-CoV-2 infected Vero E6 cells

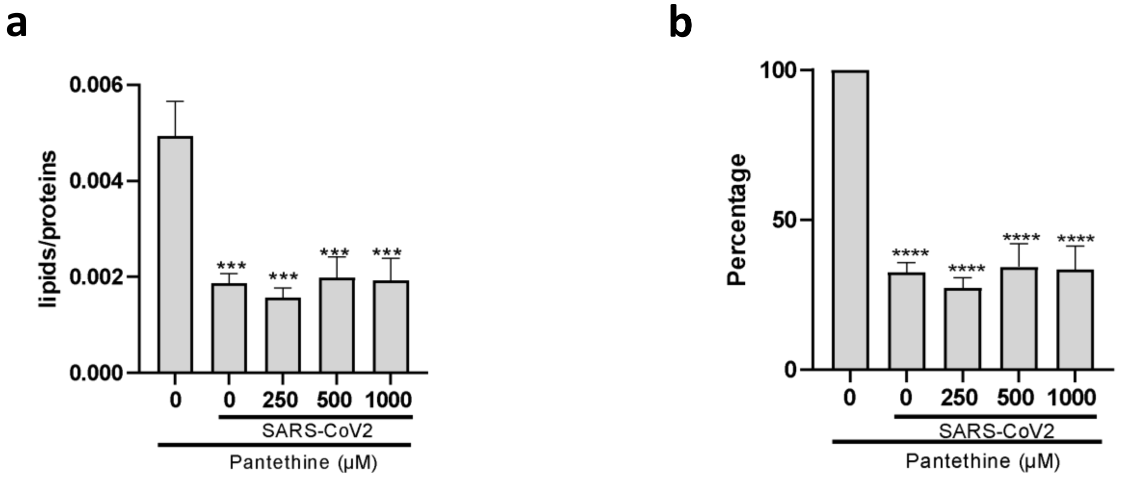

Supplementary Figure S8. Pantethine alters total cholesterol in non-infected and SARS-CoV-2 infected Vero E6 cells

Vero E6 cells non infected (**A**) or infected with SARS-CoV-2 (**B**) were incubated with different concentrations of pantethine (250 µM, 500 µM and 1000µM). After 72 hours, cells were collected, there lipids extracted and quantified. For “short time incubation” (**Aa** and **Ab**), non-infected cells were incubated with pantethine for 24 hours and then the media was changed without any more add of the drug to reach 72h. For “Long time incubation” (**Ac** and **Ad**), non-infected cells were incubated with pantethine during 72 hours without changing the media and the pantethine was added each day. Results represent mean + SEM of the ratio of total cholesterol(ng)/total proteins(ng) (**Aa and Ac**), and percentage + SEM of the data compared to the untreated control group (**Ab and Ad**), obtained from 3 independent experiments. $^{**}p < 0.01$, $^{***}p < 0.001$ and $^{****}p < 0.0001$ compared to the control group (untreated cells) by *One-way ANOVA test* followed by *Dunnett's post*-hoc *test*.

In **(B)** Vero E6 cells were incubated with different concentrations of pantethine (250 µM, 500 µM and 1000µM) for 1h, then infected with SARS-CoV-2 at MOI 0.05. For “Long time incubation”, the cells were cultured with drug-containing medium for 72 hours without removing the virus from the culture and where the pantethine was added each day. Results represent mean + SEM of the ratio of total cholesterol(ng)/total proteins(ng) (**Ba**), and percentage + SEM of the data compared to the untreated control group (**Bb**), obtained from 3 independent experiments. $^{***}p < 0.001$ and $^{****}p < 0.0001$ compared to the control group (untreated cells) by *One-way ANOVA test* followed by *Dunnett's post*-hoc *test*.

Supplementary Figure S9

Non-infected Vero E6 (A) and Calu-3a (B)

A

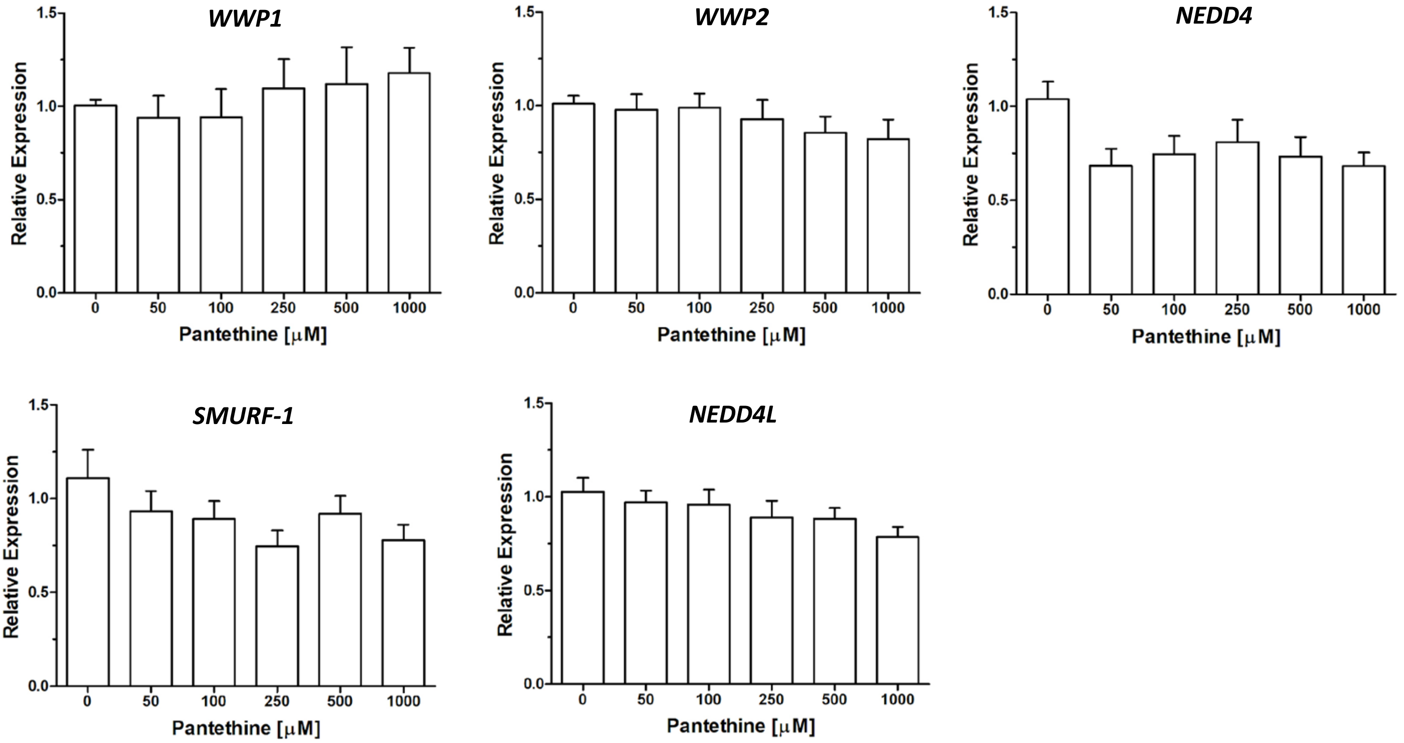

B

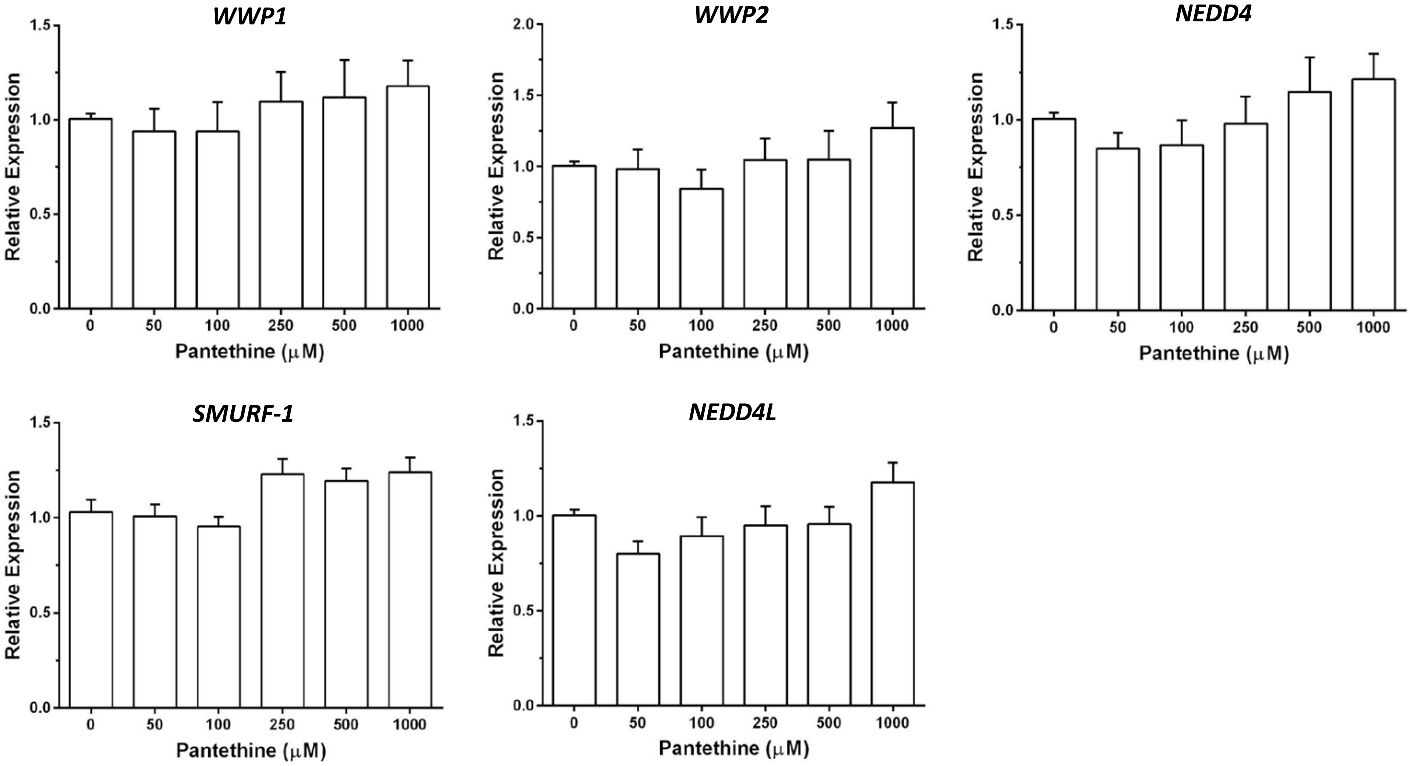

Supplementary Figure S9. Pantethine treatment had no effect on HECT E3 ligases expression in non-infected cells.

Vero E6 (**A**) and Calu-3a (**B**) cells, were treated or not with pantethine (Full-time treatment). Seventy-two (Vero E6) or forty-eight (Calu-3a) hours post-infection, cells were collected, and their RNA extracted to evaluate the expression levels of different HECT E3 ligases by qRT-PCR. Calculated Ct values were converted to fold-reduction of samples compared to the housekeeping gene *GAPDH* using the ΔΔCt method (fold changed in RNA=2^-ΔΔCt). Results represent mean + SEM, from 4 independent experiments.

Supplementary Figure S10

Non-infected Vero E6 (A) and Calu-3a (B)

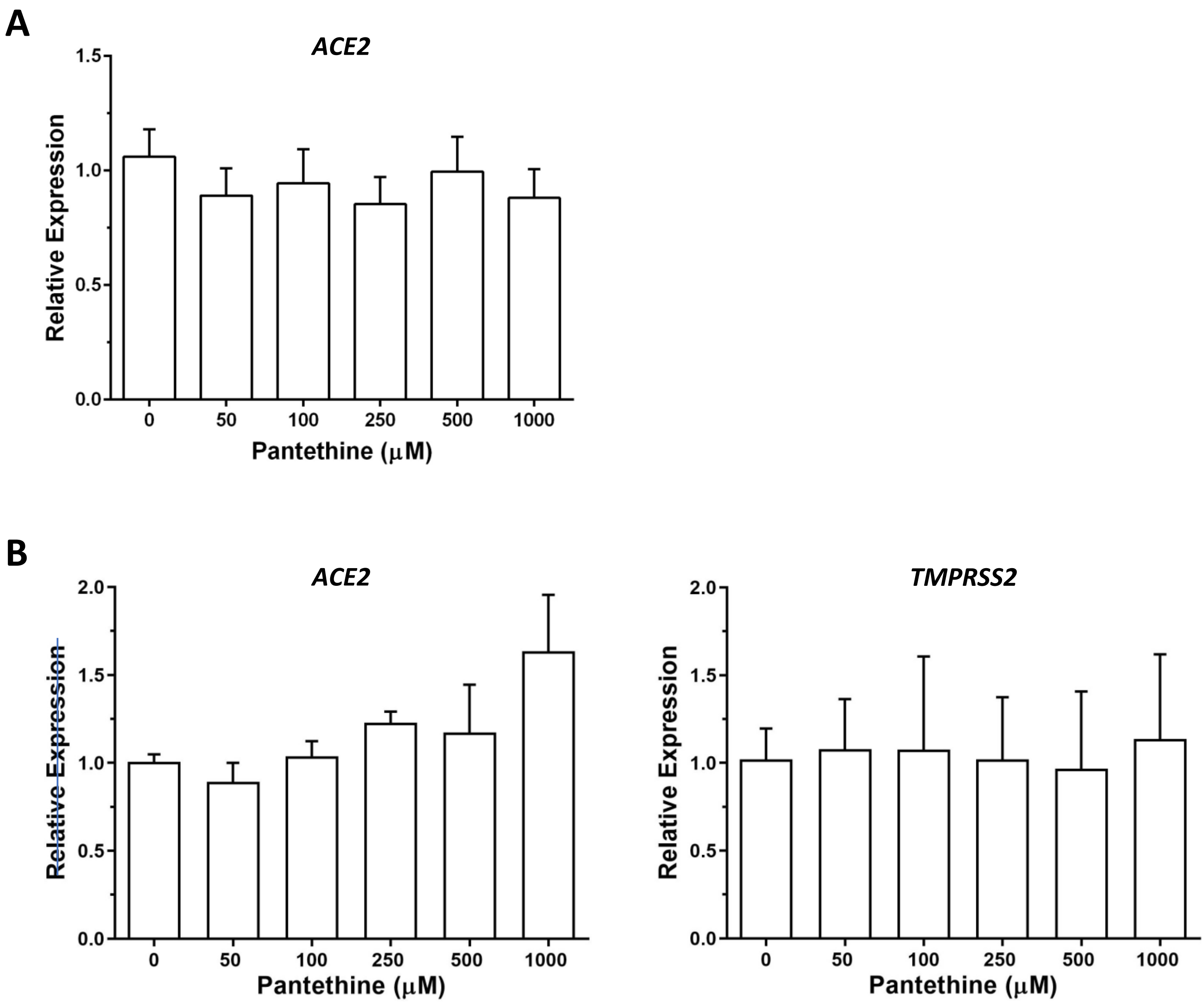

Supplementary Figure S10. Pantethine treatment had no significant effect on ACE2 and TMPRSS2 expression in non-infected cells.

Vero E6 (**A**) or Calu-3a (**B**) cells were treated or not with pantethine (Full-time treatment). Seventy-two (Vero E6) or forty-eight (Calu-3a) hours post-infection, cells were collected, and their RNA extracted to evaluate the expression levels of *ACE2* and *TMPRSS2* by qRT-PCR. Calculated Ct values were converted to fold-reduction of samples compared to the housekeeping gene *GAPDH* using the ΔΔCt method (fold changed in RNA=2^-ΔΔCt). Results represent mean + SEM, from 3 independent experiments.

Supplementary Figure S11

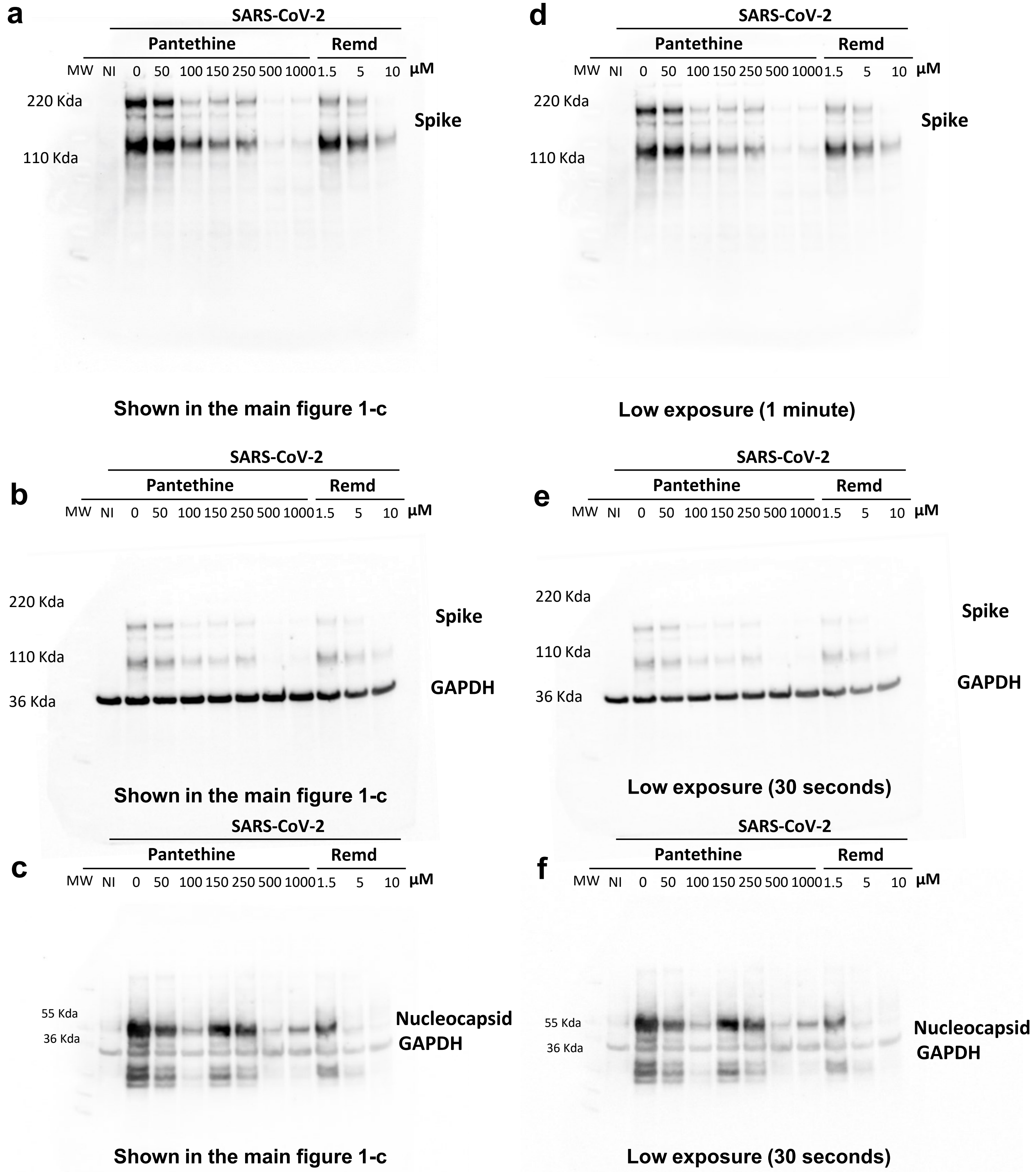

Supplementary Figure S11.

Different exposures of original and full-length blots of figure 1-C. (Full treatment-VeroE6)

The first revelation was for the Spike protein shown in the main figure (a) and its low exposure (d) , The second revelation was for GAPDH protein shown in the main figure (b) and its low exposure (e) and the third revelation was for nucleocapsid protein shown in the main figure (c) and its low exposure (f) . Remd is the abbreviation for Remdesivir

Supplementary Figure S12

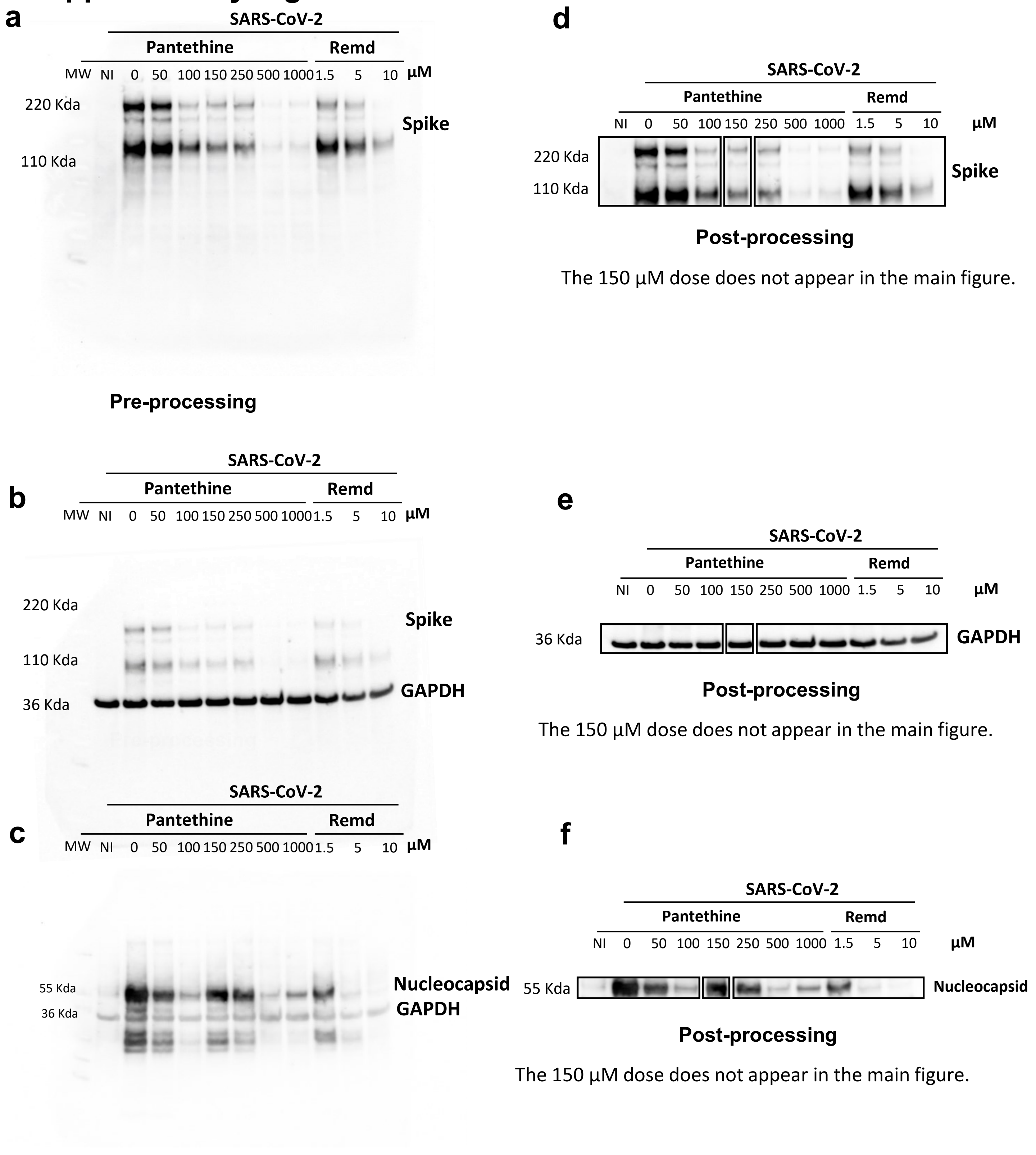

Supplementary Figure S12.

Pre and post processing images for blots of figure 1-C. (Full treatment-VeroE6)

The original image for spike protein revelation (a) and its processed version (d), The original image for the GAPDH protein revelation (b) and its processed version (e) and the original image for the nucleocapsid protein revelation (c) and its processed version (f). Remd is the abbreviation for Remdesivir

Supplementary Figure S13

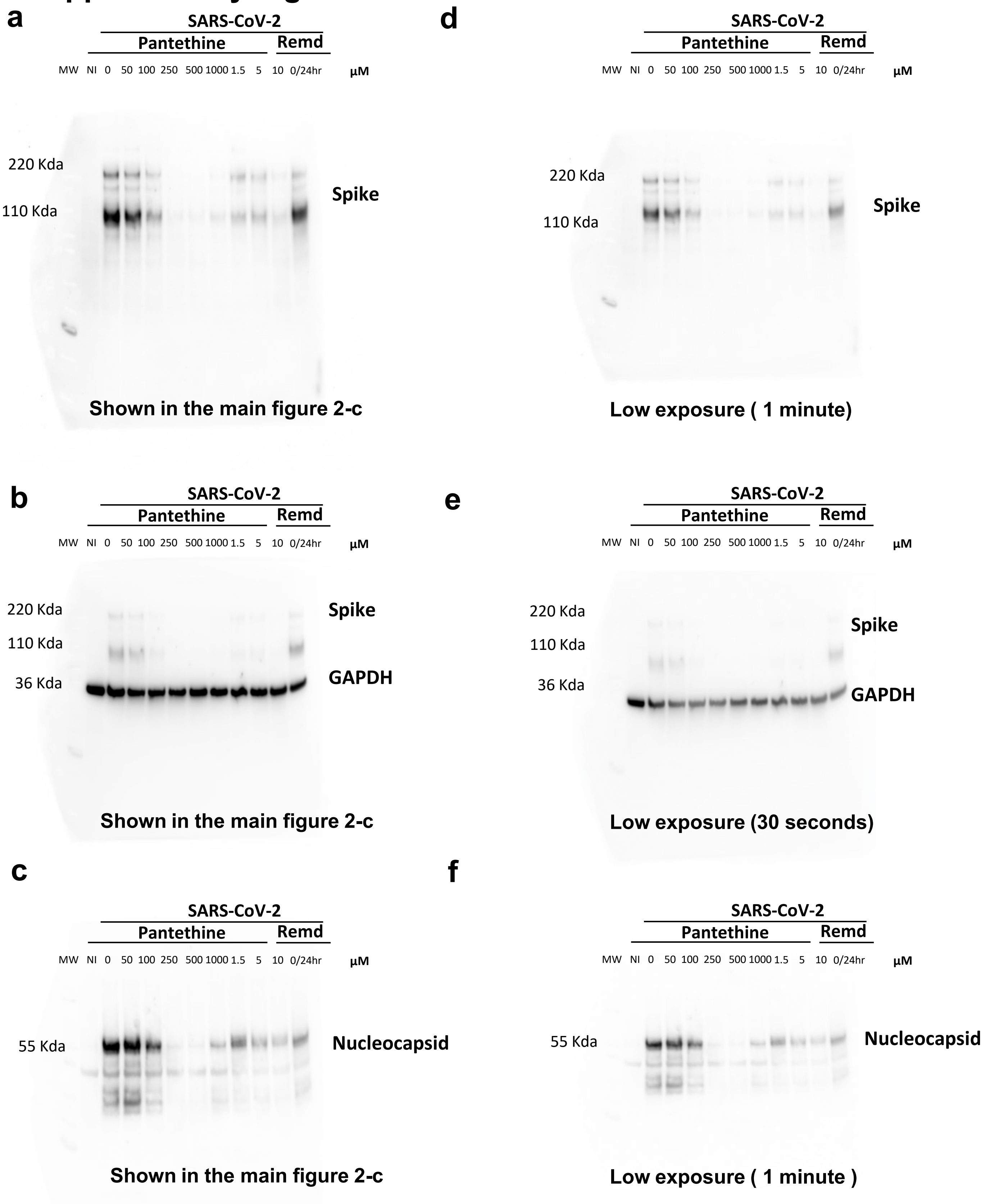

Supplementary Figure S13

Different exposures of original and full-length blots of figure 2-C. (Post entry-VeroE6)

The first revelation was for the Spike protein shown in the main figure (a) and its low exposure (d) , The second revelation was for GAPDH protein shown in the main figure (b) and its low exposure (e) and the third revelation was for nucleocapsid protein shown in the main figure (c) and its low exposure (f) . Remd is the abbreviation for Remdesivir .

Supplementary Figure S14

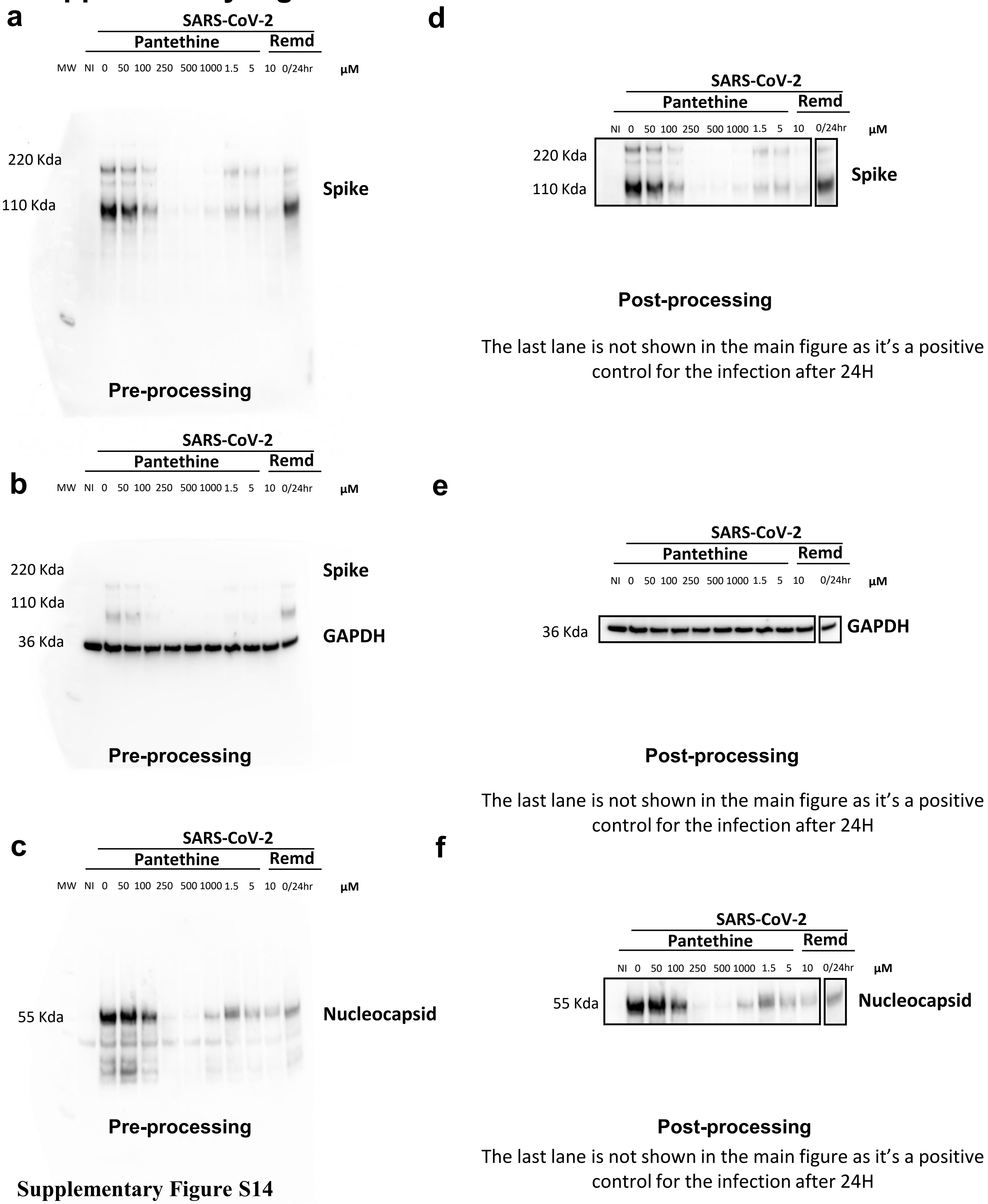

Supplementary Figure S14

Pre and post processing images for blots of figure 2-C. (Post-Entry-VeroE6)

The original image for spike protein revelation (a) and its processed version (d), the original image for the GAPDH protein revelation (b) and its processed version (e) and the original image for the nucleocapsid protein revelation (c) and its processed version (f). Remd is the abbreviation for Remdesivir

Supplementary Figure S15

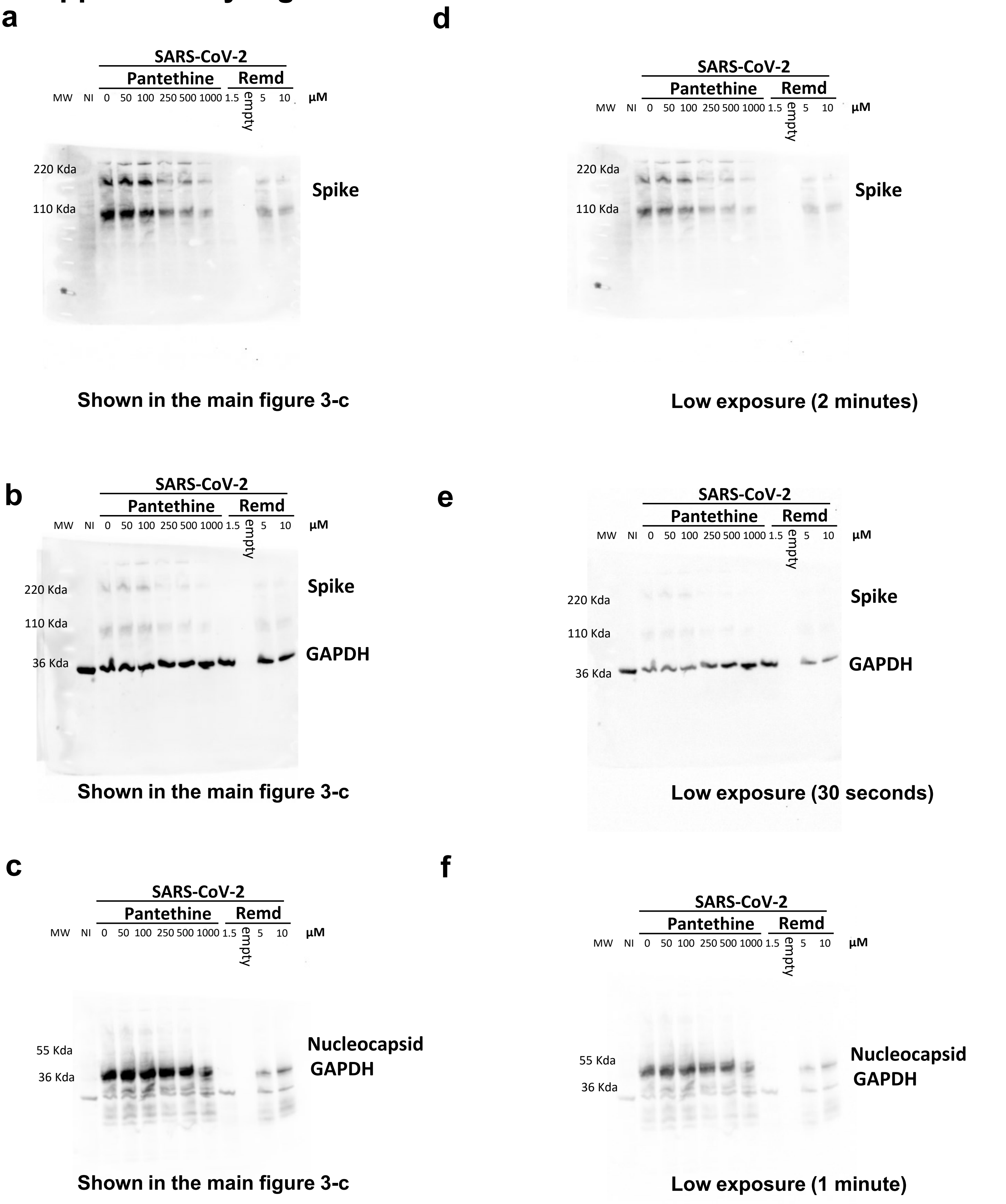

Supplementary Figure S15

Different exposures of original and full-length blots of figure 2-C. (Entry-24h--VeroE6)

The first revelation was for the Spike protein shown in the main figure (a) and its low exposure (d) , The second revelation was for GAPDH protein shown in the main figure (b) and its low exposure (e) and the third revelation was for nucleocapsid protein shown in the main figure (c) and its low exposure (f) . Remd is the abbreviation for Remdesivir .

Supplementary Figure S16

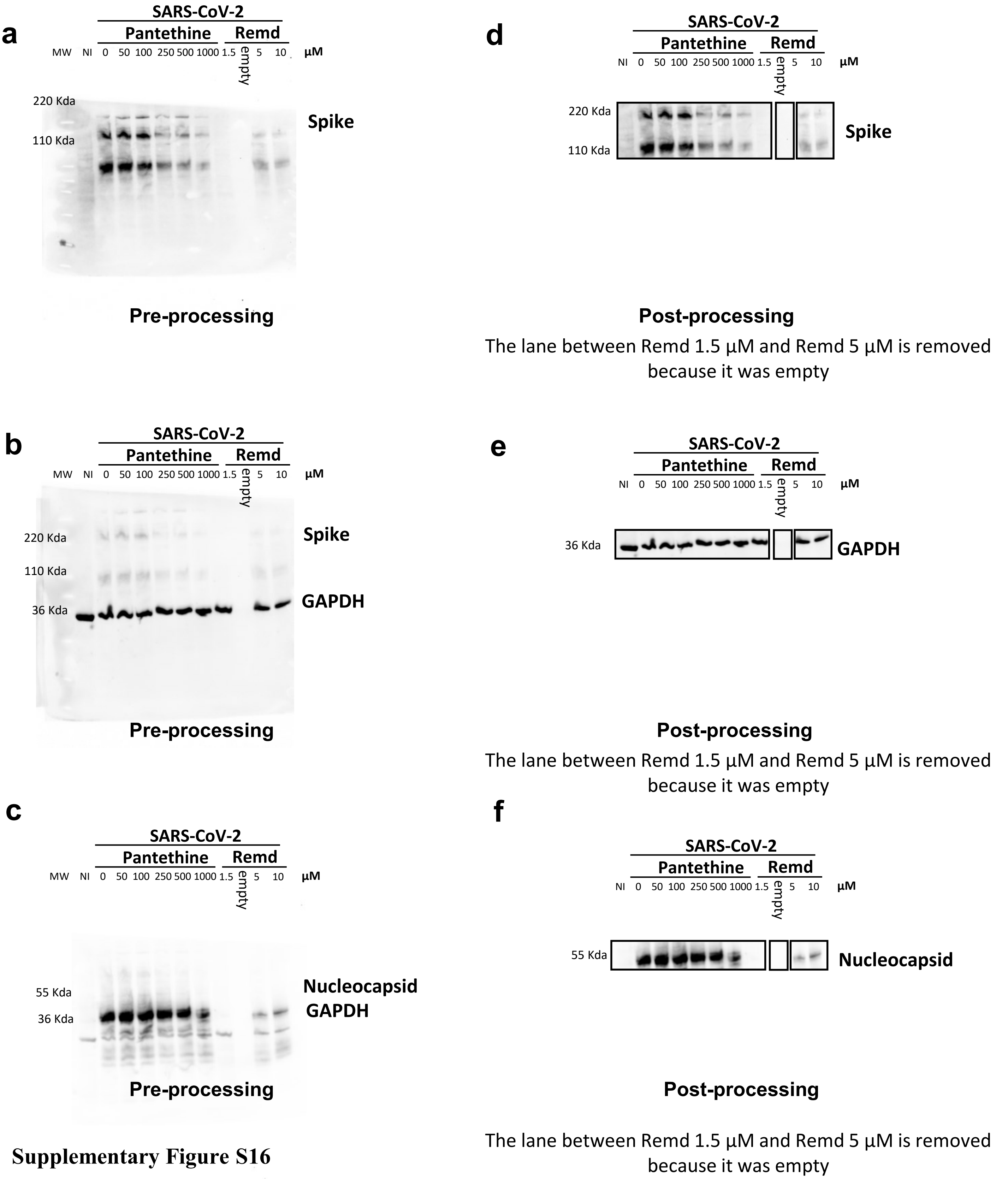

Supplementary Figure S16

Pre and post processing images for blots of figure 3-C . (Entry-24h--VeroE6)

The original image for spike protein revelation (a) and its processed version (d), The original image for the GAPDH protein revelation (b) and its processed version (e) and the original image for the nucleocapsid protein revelation (c) and its processed version (f). Remd is the abbreviation for Remdesivir

Supplementary Figure S17

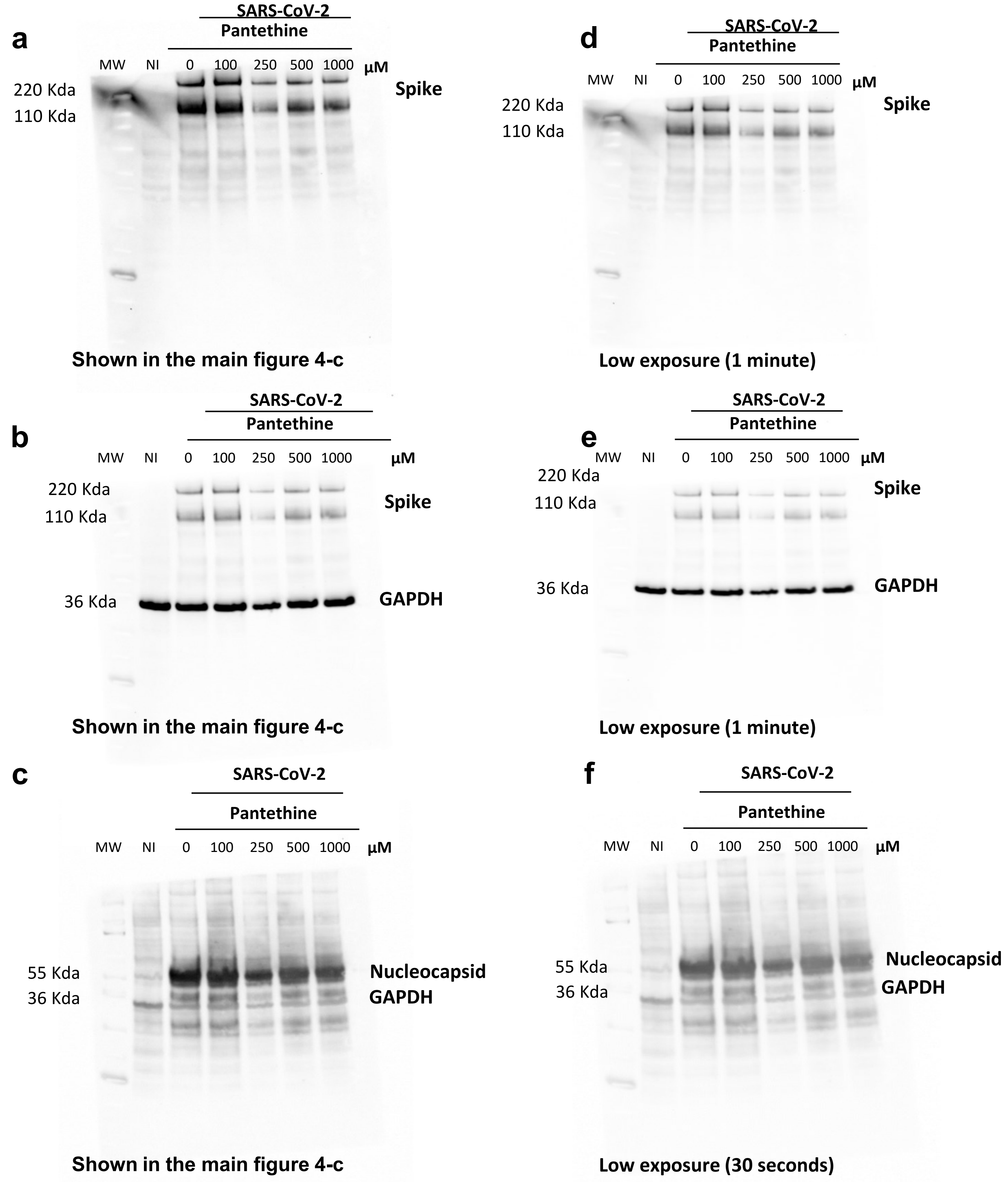

Supplementary Figure S17

Different exposures of original and full-length blots 4-C. (Full Treatment Calu-3a)

The first revelation was for the Spike protein shown in the main figure (a) and its low exposure (d) , The second revelation was for GAPDH protein shown in the main figure (b) and its low exposure (e) and the third revelation was for nucleocapsid protein shown in the main figure (c) and its low exposure (f) . Remd is the abbreviation for Remdesivir .

Supplementary Figure S18

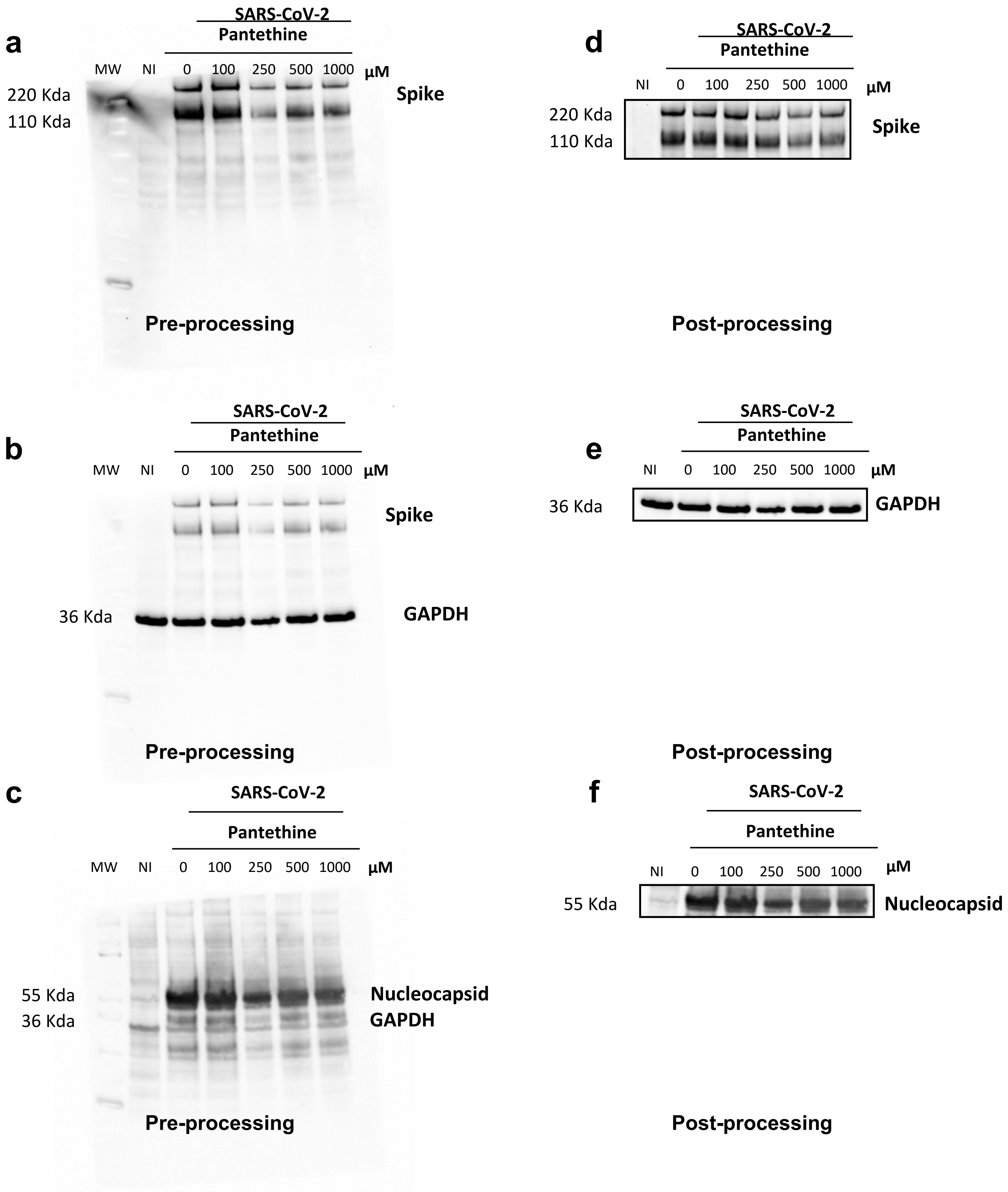

Supplementary Figure S18

Pre and post processing images for blots of figure 4-C . (Full treatment-VeroE6)

The original image for spike protein revelation (a) and its processed version (d), The original image for the GAPDH protein revelation (b) and its processed version (e) and the original image for the nucleocapsid protein revelation (c) and its processed version (f). Remd is the abbreviation for Remdesivir

Supplementary Figure S19- Original blots of Fig S3-B

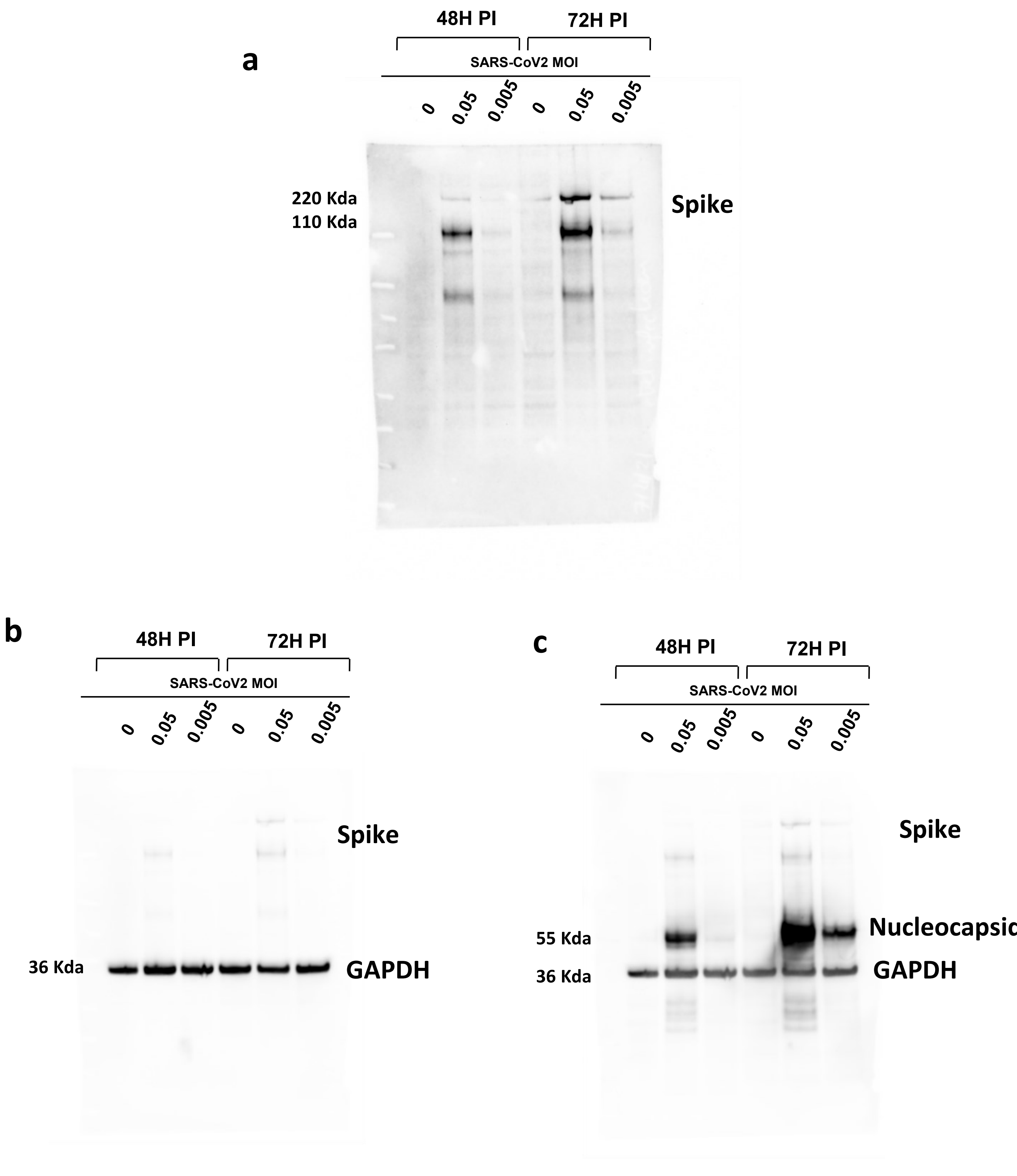

Supplementary Figure S19. Original and full-length blots of figure S3-B. The setup of infectious system with SARS-CoV-2 in Vero-E6 cells.

First revelation for the Spike protein (a), second revelation for the GAPDH (b) and third revelation for the nucleocapsid protein (c).

Supplementary Figure S20- low exposure of original blots of Fig S3-B

a

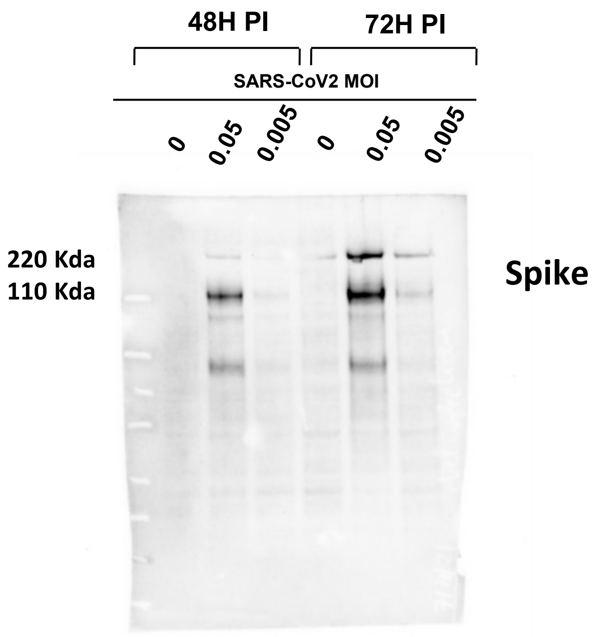

b

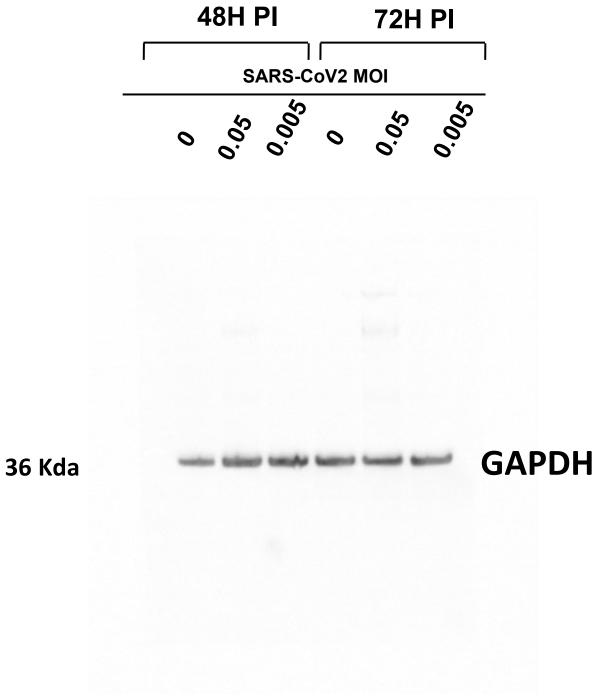

c

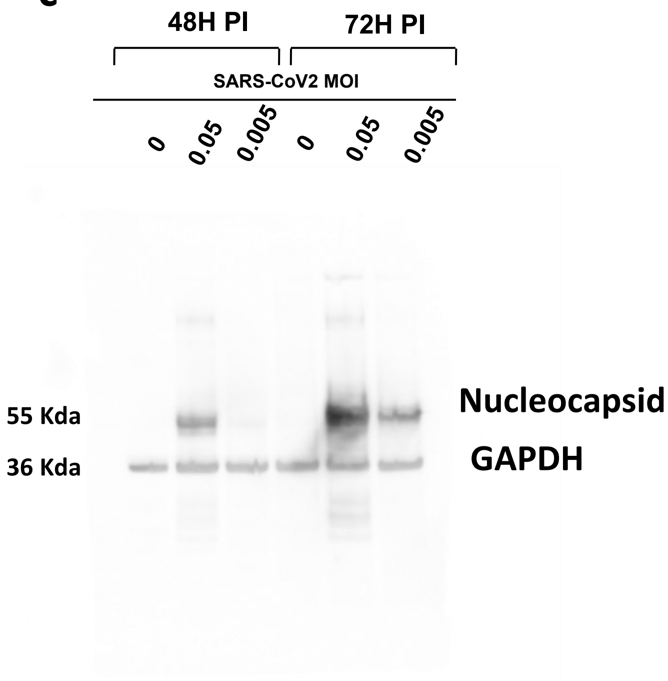

Supplementary Figure S20. Low exposure of the original and full-length blots of figure S3-B. The setup of infectious system with SARS-CoV-2 in Vero-E6 cells.

First revelation for the Spike protein (a), second revelation for the GAPDH (b) and third revelation for the nucleocapsid protein (c).
